# Genetic architecture of the structural connectome

**DOI:** 10.1038/s41467-024-46023-2

**Published:** 2024-03-04

**Authors:** Michael Wainberg, Natalie J. Forde, Salim Mansour, Isabel Kerrebijn, Sarah E. Medland, Colin Hawco, Shreejoy J. Tripathy

**Affiliations:** 1 Krembil Centre for Neuroinformatics, Centre for Addiction and Mental Health, Toronto, ON Canada; 2 Prosserman Centre for Population Health Research, Lunenfeld-Tanenbaum Research Institute, Sinai Health, Toronto, ON Canada; 3 Department of Psychiatry, University of Toronto, Toronto, ON Canada; 4 Institute of Medical Sciences, University of Toronto, Toronto, ON Canada; 5 Department of Cognitive Neuroscience, Donders Institute for Brain, Cognition and Behaviour, Radboud University Nijmegen Medical Centre, Nijmegen, The Netherlands; 6 Campbell Family Mental Health Research Institute, Centre for Addiction and Mental Health, Toronto, ON Canada; 7 QIMR Berghofer Medical Research Institute, Brisbane, QLD Australia; 8 School of Psychology, University of Queensland, Brisbane, QLD Australia; 9 Faculty of Medicine, University of Queensland, Brisbane, QLD Australia; 10 Department of Physiology, University of Toronto, Toronto, ON Canada

**Keywords:** Genome-wide association studies, Genetics of the nervous system

## Abstract

Myelinated axons form long-range connections that enable rapid communication between distant brain regions, but how genetics governs the strength and organization of these connections remains unclear. We perform genome-wide association studies of 206 structural connectivity measures derived from diffusion magnetic resonance imaging tractography of 26,333 UK Biobank participants, each representing the density of myelinated connections within or between a pair of cortical networks, subcortical structures or cortical hemispheres. We identify 30 independent genome-wide significant variants after Bonferroni correction for the number of measures studied (126 variants at nominal genome-wide significance) implicating genes involved in myelination (*SEMA3A*), neurite elongation and guidance (*NUAK1*, *STRN*, *DPYSL2*, *EPHA3*, *SEMA3A*, *HGF*, *SHTN1*), neural cell proliferation and differentiation (*GMNC*, *CELF4*, *HGF*), neuronal migration (*CCDC88C*), cytoskeletal organization (*CTTNBP2*, *MAPT*, *DAAM1*, *MYO16*, *PLEC*), and brain metal transport (*SLC39A8*). These variants have four broad patterns of spatial association with structural connectivity: some have disproportionately strong associations with corticothalamic connectivity, interhemispheric connectivity, or both, while others are more spatially diffuse. Structural connectivity measures are highly polygenic, with a median of 9.1 percent of common variants estimated to have non-zero effects on each measure, and exhibited signatures of negative selection. Structural connectivity measures have significant genetic correlations with a variety of neuropsychiatric and cognitive traits, indicating that connectivity-altering variants tend to influence brain health and cognitive function. Heritability is enriched in regions with increased chromatin accessibility in adult oligodendrocytes (as well as microglia, inhibitory neurons and astrocytes) and multiple fetal cell types, suggesting that genetic control of structural connectivity is partially mediated by effects on myelination and early brain development. Our results indicate pervasive, pleiotropic, and spatially structured genetic control of white-matter structural connectivity via diverse neurodevelopmental pathways, and support the relevance of this genetic control to healthy brain function.

## Abstract (web summary)

The structural connectome is the complete set of anatomical connections between brain cells. Here, the authors perform a genome-wide association study of white-matter structural connectivity in the human brain, finding 30 variants influencing the density of myelinated connections between brain regions.

## Introduction

The human brain’s volume is about equal parts gray matter and white matter. Unlike gray matter, which mainly contains densely packed cell bodies of neurons and glia, white matter is almost exclusively made up of bundles of myelinated axons^1^. The primary purpose of myelin is to considerably speed up action potential conduction along axons, from 0.5–10 m/s to ~150 m/s^2^, enabling axons (nerve fibers) traveling through the white matter to efficiently transmit information between distant brain regions. White matter fibers are key constituents of the brain’s structural connectome, the complete set of anatomical connections between brain cells^3^. Structural connectivity is a fundamental organizational property of the brain^3,4^.

Microstructural properties of the white matter can be quantified non-invasively in vivo via diffusion magnetic resonance imaging (dMRI). This is because the myelin around an axon, as well as the axonal membranes themselves and the coherence or density (organization) of axons within fiber bundles, constitute physical barriers that restrict the diffusion of water molecules, so that they diffuse more readily along the axon than perpendicular to it, resulting in a distortion of the MRI signal when a directional magnetic field gradient is applied^5–7^.

Efforts such as The Enhancing Neuroimaging Genetics through Meta-analysis (ENIGMA) Consortium^8^ and the UK Biobank^9^ have gathered large-scale cohorts with both brain MRI and genetic data. Genome-wide association studies (GWAS) of MRI phenotypes^10–26^ based on these efforts have elucidated the genetic architecture of brain structure and function with unprecedented detail. Most white-matter GWAS to date^13,14,18,19^ have relied upon tract-based spatial statistics (TBSS) (13), averaging white matter microstructural properties across entire tracts, such as the corpus callosum. TBSS effectively indexes general white-matter health^27^, but does not explicitly consider how brain regions connect to each other.

Structural connectivity can be evaluated using a technique known as fiber tracking or tractography^28–30^, which constructs a model of the paths of white matter fibers based on the directionality of water diffusion through the white matter. By overlaying a brain atlas of one’s choice, the connectivity between pairs of brain regions can be quantified. Thus, unlike TBSS, tractography has the ability to quantify the structural connectome, providing rich detail on the whole-brain organizational properties relating to complex processes such as cognition^31^ and functional connectivity^32^. Tractography has implicated connectomic disturbances in diverse neuropsychiatric disorders^33^, including schizophrenia^34–46^, bipolar disorder^41,44,47,48^, major depressive disorder^49^, attention-deficit hyperactivity disorder^50–52^, autism^53–55^, Alzheimer’s disease^56–58^, and multiple sclerosis^59–66^.

**Fig. 1:**
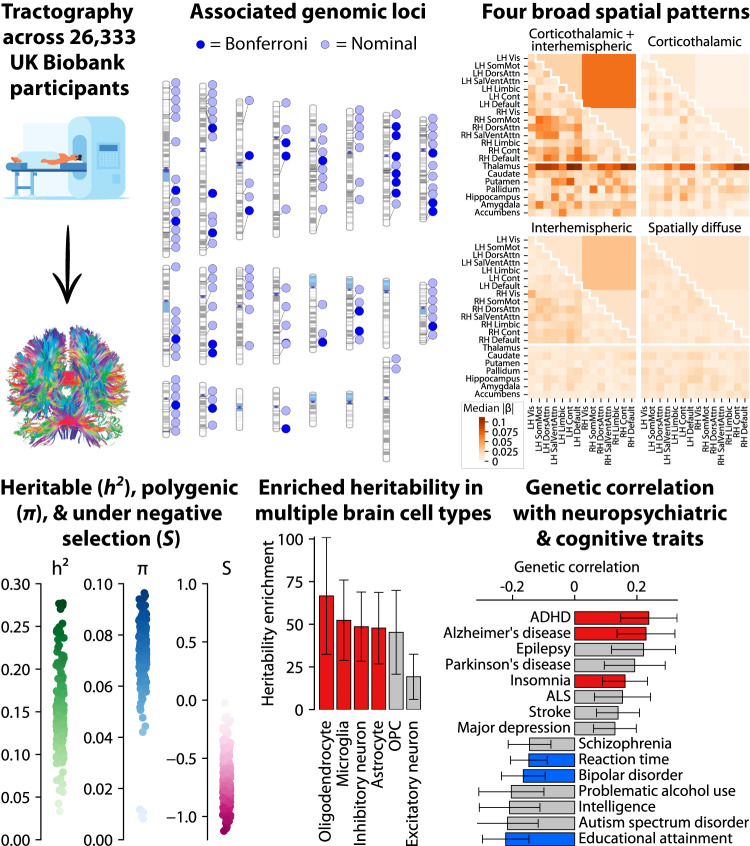
Study overview. Top left: Measurement of tractograms from participants with brain imaging data in the UK Biobank. Top middle: genomic locations of common genetic variants associated with structural connectivity, with chromosomes in ascending order (first row: chr1-8; second row: chr9-16; third row: chr17-22 and chrX). 30 independent variants were genome-wide significant after Bonferroni correction for the 206 structural connectivity measures studied (dark blue), and 96 more reached nominal genome-wide significance (light blue). Top right: the 30 variants cluster into four broad patterns of spatial association: corticothalamic and interhemispheric (3 variants), corticothalamic only (1 variant), interhemispheric only (8 variants), and spatially diffuse (18 variants). Heatmaps denote the median effect size magnitude (|β | ) of variants with each pattern on each of the 206 structural connectivity measures. Bottom left: heritability (*h*^*2*^), polygenicity (*π*), and selection parameter (*S*) for each of the 206 measures. Bottom middle and right: heritability enrichments for regions with increased chromatin accessibility in each of 6 adult brain cell types, and genetic correlations with 15 brain-related traits. Bars show maximums across the 206 measures; red/blue bars are significant after Bonferroni correction. OPC oligodendrocyte progenitor cell, ADHD attention-deficit/hyperactivity disorder, ALS amyotrophic lateral sclerosis, disord. disorder. Error bars represent 95% confidence intervals.

Here, we expand upon prior white matter GWAS to examine 206 tractography-based structural connectivity measures representing the rich organizational structure of the brain. Each measure quantifies the density of white matter fibers within or between a pair of well-established large-scale cortical brain networks^67^, subcortical structures, or hemispheres. Our primary analysis (summarized in Fig. 1) encompasses 26,333 participants from the UK Biobank with diffusion MRI scans – two orders of magnitude larger than the only prior GWAS of structural connectivity^68^, which studied 366 participants.

## Results

### Tractography across 26,333 UK Biobank participants

**Fig. 2:**
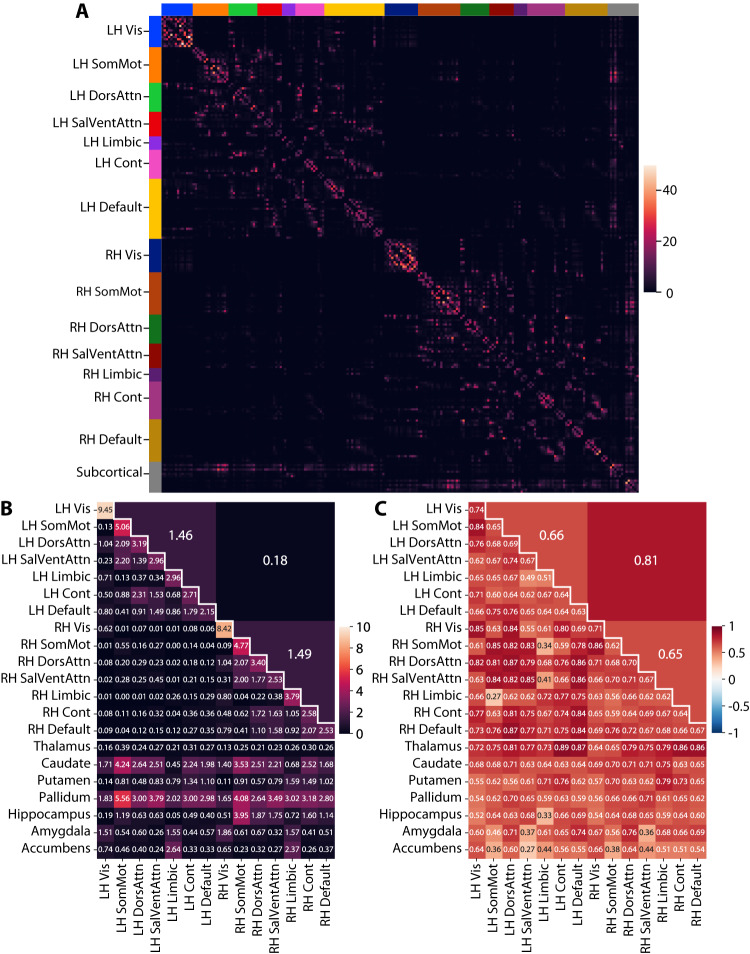
Tractography across 26,333 UK Biobank participants. **A** Participant-averaged connectivity between each pair of parcels in the 200-parcel Schaefer cortical atlas + 14 subcortical parcels from the Harvard-Oxford atlas (in order: left thalamus, left caudate, left putamen, left pallidum, left hippocampus, left amygdala, left accumbens, right thalamus, right caudate, right putamen, right pallidum, right hippocampus, right amygdala, right accumbens). Heatmap entries indicate the weighted number of streamlines connecting each pair of parcels, averaged across participants (see “Tractography pipeline”, “Methods”). Color bands along the top and left indicate Yeo 7 networks. LH left-hemisphere, RH right-hemisphere, Vis visual, SomMot somatomotor, DorsAttn dorsal attention, SalVentAttn salience/ventral attention, Limbic limbic, Cont control, Default default mode. **B** Participant averages of the 206 connectivity measures. The three large tiles above the diagonal represent hemisphere-level measures. **C** Inter-replicate type 3 intraclass correlation coefficients (ICCs) across 2400 participants with replicate MRI scans, calculated using the *intraclass_corr* function from the *pingouin* Python package^200^.

We established a tractography pipeline to infer white-matter structural connectomes from 26,333 UK Biobank participants (2400 with replicate scans, which were not used for the GWAS) with structural and diffusion MRI scans passing quality control (see “Cohort selection and MRI quality control”, Methods). Participants were 53% female and aged 40–70 (median 55) at the time of their first scan. dMRI data were extensively pre processed according to the UKB pipelines prior to use (biobank.ctsu.ox.ac.uk/crystal/ukb/docs/brain_mri.pdf). To obtain connectomes for each participant, we used the MRtrix3 diffusion MRI software package^69^ to: (1) estimate a fiber orientation distribution via multi-shell multi-tissue constrained spherical deconvolution of the diffusion MRI scan^70^; (2) perform anatomically constrained probabilistic tractography via second-order integration^71^, selecting 1 million streamlines seeded from the gray/white matter interface; (3) weight each streamline via the SIFT2 algorithm^72^ so that the density of reconstructed connections better reflects the density of the underlying white matter fibers; and (4) generate a symmetric 214 × 214 connectome matrix (Fig. 2A) denoting the density of streamlines connecting each pair of parcels in the 200-parcel Schaefer cortical atlas^73^ plus 14 subcortical parcels from the Harvard-Oxford atlas (Supplementary Data 4), scaled by the average length of the connecting streamlines and divided by the product of the two parcel volumes.

Rather than performing 22,791 GWAS, one for each pair of parcels, we opted to reduce the dimensionality of each participant’s connectome matrix in a biologically interpretable way. We derived three types of measures, for a total of 206 measures (Fig. 2B): (1) hemisphere-level cortical-to-cortical connectivity (3 measures: left intra-hemisphere, right intra-hemisphere and inter-hemisphere), (2) network-level cortical-to-cortical connectivity within and between each of the 14 hemisphere-specific “Yeo 7” networks^67^ (105 measures, e.g. left-hemisphere visual, “LH Vis”, to right-hemisphere somatomotor, “RH SomMot”), and (3) cortical-to-subcortical connectivity between each of these 14 “Yeo 7” networks and 7 subcortical structures: thalamus, caudate, putamen, pallidum, hippocampus, amygdala, and accumbens (98 measures). These 206 measures were moderately replicable (median scan-rescan intraclass correlation coefficient = 0.67) across the 2400 participants with replicate scans, taken 1.0–5.3 years (median 2.2) after the original scan (Fig. 2C).

### Variants associated with structural connectivity

**Fig. 3:**
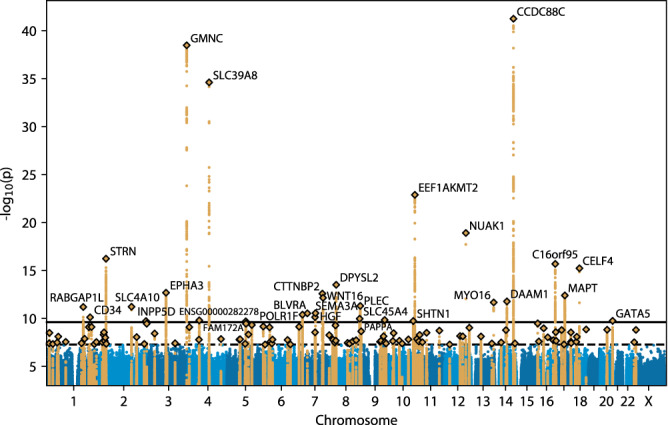
Manhattan plot of each variant’s minimum *p* value across the 206 structural connectivity measures. Gold variants indicate linkage disequilibrium (LD) clumps passing nominal genome-wide significance (dashed black line); black diamonds highlight the lead variant in each clump. Nearest genes are labeled for each clump passing Bonferroni-corrected genome-wide significance (solid black line).

We performed genome-wide association studies on these 206 connectivity measures using the regenie genetic analysis toolkit^74^. We analyzed 9,423,516 variants present in the UK Biobank’s imputed genotypes (imputed to a combination of the Haplotype Reference Consortium^75^, UK10K^76^ and 1000 Genomes Phase 3^77^ cohorts) which had at least 1% minor allele frequency and passed quality control (see Methods). We covaried for age, sex, age × sex, age^2^, age^2^ × sex, genotyping array, scanner site (*n* = 22 sites), total intracranial volume, and the top 10 genotype principal components. We identified 30 genome-wide significant loci (Table 1, Fig. 1, Fig. 3) associated with at least one of the 206 measures after Bonferroni correction for the number of measures tested (i.e. at *p* < 5 × 10^–8^ / 206 ≈ 2.4 × 10^–10^). We identified 126 loci (Fig. 1, Fig. 3, Supplementary Data 1) at the less stringent threshold of nominal genome-wide significance (*p* < 5 × 10^–8^). In a replication analysis of these 126 loci in 665 participants of non-European genetic ancestry, 75 of the 126 lead variants had at least 1% frequency and passed quality control, and these variants were 2.7 times more likely to have the same direction of effect than expected by chance (Fisher *p* = 0.038; Supplementary Data 2). The one association from the prior structural connectivity GWAS mentioned in the introduction^68^, the intronic variant rs2618516 in *SPON1*, failed to replicate in the European cohort (uncorrected *p* > 0.006 for all 206 measures).

**Table 1:** The 30 genome-wide significant loci after Bonferroni correction for the number of measures studied

Variant	Location (hg19)	A1	A2	A1 freq.	# mea- sures	Most significantly associated measure	Effect size	*p* value	Nearest gene(s)	Candidate causal gene(s)
rs941760	chr14:91,881,751	T	C	44%	8	RH Cont to thalamus	–0.10	5.9 × 10^–42^	*CCDC88C*	*CCDC88C*
rs905124	chr3:190,657,360	A	T	38%	21	RH Cont to thalamus	0.10	3.5 × 10^–39^	*GMNC*	*GMNC*
rs13107325	chr4:103,188,709	T	C	7%	7	RH Limbic to hippocampus	–0.20	2.5 × 10^–35^	*SLC39A8*	*SLC39A8*
rs11245366	chr10:126,482,849	T	C	56%	2	LH Vis to RH Vis	–0.08	1.3 × 10^–23^	*EEF1AKMT2*	*--*
rs12146713	chr12:106,476,805	C	T	10%	12	LH Cont to thalamus	–0.11	1.2 × 10^–19^	*NUAK1*	*NUAK1*
rs35050623	chr2:37,063,240	CT	C	54%	5	LH Vis to RH Vis	–0.06	5.7 × 10^–17^	*STRN*	*STRN*
rs4843550	chr16:87,236,383	C	G	57%	4	RH Limbic to putamen	–0.06	2.0 × 10^–16^	*C16orf95*	*--*
rs4799450	chr18:35,028,901	C	T	64%	2	RH Vis to pallidum	0.07	5.7 × 10^–16^	*CELF4*	*CELF4*
rs199790004	chr8:26,427,757	A	AG	62%	1	LH Default to putamen	0.06	3.1 × 10^–14^	*DPYSL2*	*DPYSL2*
rs35124509	chr3:89,521,693	C	T	39%	3	LH Vis to LH Cont	–0.06	2.0 × 10^–13^	*EPHA3*	*EPHA3*
rs150346963	chr7:117,625,599	T	C	41%	1	LH Default to RH Limbic	0.06	2.5 × 10^–13^	*CTTNBP2*	*CTTNBP2*
rs118087478	chr17:44,051,589	G	T	22%	1	LH Limbic to caudate	–0.07	3.9 × 10^–13^	*MAPT*	*MAPT*
rs142005327	chr7:120,969,969	GCT	G	26%	2	LH SalVentAttn to LH Cont	0.06	6.9 × 10^–13^	*WNT16*	*--*
rs79814107	chr14:59,637,503	G	A	12%	1	LH Cont to RH Vis	0.08	1.6 × 10^–12^	*DAAM1*	*DAAM1*
rs34844662	chr13:109,687,510	AT	A	34%	1	LH Vis to RH Vis	–0.06	2.1 × 10^–12^	*MYO16*	*MYO16*
rs6558407	chr8:144,995,494	T	C	43%	5	LH SalVentAttn to RH SomMot	0.05	4.9 × 10^–12^	*PLEC*	*PLEC*
rs59154421	chr2:162,813,034	C	CTG	29%	1	LH Limbic to RH Default	–0.06	6.0 × 10^–12^	*SLC4A10*	*SLC4A10*
rs75650221	chr1:174,421,994	T	C	4%	1	LH Default to caudate	0.13	6.3 × 10^–12^	*RABGAP1L*	*--*
rs13228652	chr7:83,761,158	C	G	18%	1	LH Vis to RH Vis	0.07	2.8 × 10^–11^	*SEMA3A*	*SEMA3A*
rs141628902	chr7:43,818,855	T	C	3%	1	LH SalVentAttn to amygdala	0.16	2.9 × 10^–11^	*BLVRA*	*--*
rs1158069	chr7:19,643,101	A	G	37%	1	RH Vis to caudate	–0.05	4.0 × 10^–11^	*POLR1F*	*--*
rs975360	chr7:81,411,028	C	T	40%	1	RH Cont to amygdala	–0.05	7.2 × 10^–11^	*HGF*	*HGF*
rs2796245	chr1:208,030,852	A	G	86%	1	Cross-hemisphere	0.06	7.4 × 10^–11^	*CD34*	*--*
rs1865249	chr8:142,215,982	C	A	61%	1	LH Cont to RH Cont	0.05	1.1 × 10^–10^	*SLC45A4*	*--*
rs415978	chr9:118,985,910	A	G	40%	1	RH SomMot to caudate	0.05	1.4 × 10^–10^	*PAPPA*	*--*
rs1391762	chr4:54,755,157	G	A	82%	1	LH SomMot to RH SomMot	–0.06	1.6 × 10^–10^	*ENSG00000282278*	*--*
rs6062264	chr20:61,154,871	T	C	28%	1	RH Vis to RH Vis	–0.05	1.8 × 10^–10^	*GATA5*	*--*
rs5788199	chr10:118,649,704	TA	T	75%	1	LH DorsAttn to amygdala	0.06	1.8 × 10^–10^	*ENO4, SHTN1*	*SHTN1*
rs551746431	chr2:234,112,457	C	*	2%	1	LH Cont to thalamus	–0.20	1.8 × 10^–10^	*INPP5D*	*--*
rs115136616	chr5:93,021,116	T	C	5%	1	RH Vis to RH SomMot	0.12	2.0 × 10^–10^	*FAM172A*	*--*

Freq. = frequency; # measures refers to the number of measures (out of 206) the variant is genome-wide significant for. Effect sizes are oriented with respect to the minor allele (A1). Candidate causal genes were chosen based on a literature review of the nearest genes to each lead variant. rs59154421 (*SLC4A10*) is referred to by its UK Biobank variant identifier 2:162813034_CTG_C in the Supplementary Data and summary statistics, while rs551746431 (*INPP5D*) is referred to by its UK Biobank ID 2:234112457_CCAGCTACTCCCAAGTAGTCT_C (the reference allele, CCAGCTACTCCCAAGTAGTCT, is indicated with an asterisk in Table 1 for brevity).

### Two broad patterns of spatial association with structural connectivity

**Fig. 4:**
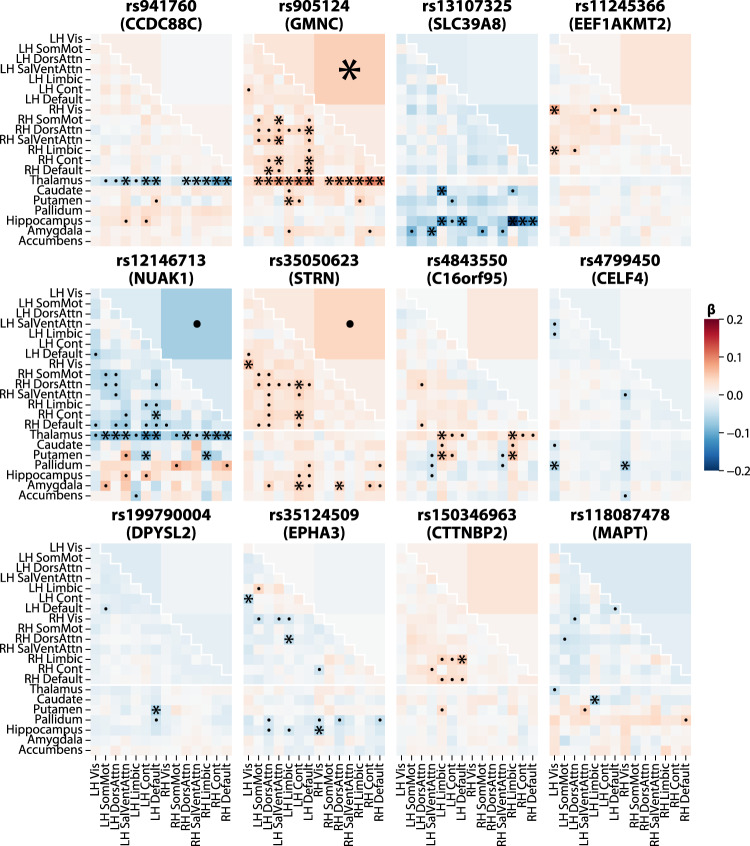
Spatial patterns of association with structural connectivity. For brevity, only the 12 most significant lead variants are shown; the remaining 18 are shown in Supplementary Fig. 1. Asterisks (*) indicate associations passing Bonferroni-corrected genome-wide significance (*p* < 5 × 10^–8^ / 206 ≈ 2.4 × 10^−10^), while dots (·) indicate associations significant after Bonferroni correction for just the 30 variants times the number of measures (*p* < 0.05 / 30 / 206 ≈ 8.1 × 10^–6^). Effect sizes (β) are oriented with respect to the minor allele, so positive effect sizes (red) indicate that the minor allele is associated with increased structural connectivity, while negative effect sizes (blue) indicate that the minor allele is associated with decreased connectivity. LH left-hemisphere, RH right-hemisphere, Vis visual, SomMot somatomotor, DorsAttn dorsal attention, SalVentAttn salience/ventral attention, Limbic limbic, Cont control, Default default mode.

We next explored the spatial pattern of association of each of the 30 lead variants across the 206 measures (Fig. 4, Supplementary Fig. 1). We annotated associations passing Bonferroni-corrected genome-wide significance (*p* < 5 × 10^–8^ / 206 ≈ 2.4 × 10^–10^) with asterisks (*). We characterized the pleiotropic effects of these 30 loci on the 206 measures, adopting a Bonferroni correction for just the 30 variants times the number of measures (*p* < 0.05 / 30 / 206 ≈ 8.1 × 10^–6^), indicated with a dot (·) in Fig. 4. At this relaxed threshold, we observed substantial pleiotropy of our 30 lead variants across the 206 measures. The median lead variant was associated with 7.5 of the 206 measures, and the most pleiotropic variant, rs2796245 (near *CD34*), was associated with 53.

We observed four broad patterns of spatial association with structural connectivity (Fig. 1). The most significant of the 30 lead variants, near *CCDC88C*, was almost exclusively associated with corticothalamic connectivity, i.e. connectivity between various cortical networks and the thalamus. Three others, near *GMNC*, *NUAK1*, and *INPP5D*, were primarily associated with inter-hemisphere cortical connections as well as corticothalamic ones (even though in the case of *INPP5D*, no inter-hemisphere connections reached significance). This inter-hemisphere pattern could exist in part because inter-hemisphere connections are more reliably detected, as reflected by their higher replicability (Fig. 2C). Eight others, near *EEF1AKMT2*, *STRN*, *CTTNBP2*, *MYO16*, *PLEC*, *SLC4A10*, *SLC45A4*, and *ENSG00000282278*, showed the inter-hemisphere pattern but not the corticothalamic one. The remaining 18 variants tended to have spatially diffuse patterns of association.

Other, more subtle patterns were also present. For instance, the *SLC39A8* variant rs13107325, uniquely among the 30 variants, was markedly associated with cortico-hippocampal, and to a lesser extent cortico-amygdalar, connectivity relative to all other measures. *SLC39A8*’s unique pattern of association may reflect its unique mechanism as a brain metal transporter (see below).

### Associated variants implicate genes with neurodevelopmental roles

17 of the 30 lead variants had nearest genes with direct biological relevance to neurodevelopment. The most significant association was with rs941760 (chr14:91,881,751), an intronic variant in *CCDC88C*. *CCDC88C* loss-of-function variants disrupt Wnt signaling, a core developmental signaling pathway^78^, and cause autosomal recessive hydrocephalus, sometimes accompanied by periventricular neuronal heterotopias (brain malformations resulting from abnormal neuronal migration), developmental delay and seizures^79–81^. (Another of the 30 lead variants has *WNT16*, a member of the Wnt family, as the nearest gene, but the relevance of *WNT16* to neurodevelopment has not been conclusively shown.)

The second-most significant association was with an intergenic variant, rs905124 (chr3:190,657,360), with *GMNC* as the nearest protein-coding gene. GMNC, also known as GEMC1, is a master regulator of multiciliated cell differentiation^82^, and in particular of the multiciliated ependymal cells that line the brain’s ventricles. GMNC is necessary for radial glial cells in the subventricular zone (one of the two main brain regions where adult neurogenesis occurs) to differentiate into multiciliated ependymal cells; when GMNC is downregulated, these radial glial cells tend to instead differentiate into neural stem cells, promoting neurogenesis^83,84^. GMNC is associated with congenital hydrocephalus (buildup of cerebrospinal fluid in the ventricles) in both humans and mice^84^. rs905124 has been previously associated with brain volume and cortical surface area according to the GWAS Catalog^85^.

The third-most significant variant, rs13107325 (chr4:103,188,709), is a missense variant in *SLC39A8* (A391T). *SLC39A8* encodes a transmembrane transporter of zinc, cadmium, iron and manganese ions sometimes referred to as ZIP8^86^. SLC38A8/ZIP8 deficiency impairs manganese uptake and compromises the function of manganese-dependent enzymes, including those required for glycosylation, leading to severe neurodevelopmental defects and other phenotypes^87^. This variant was the eighth-most significant lead variant in the largest schizophrenia GWAS to date^88^, and this variant has also been shown to impair glycosylation in the mouse brain^89^. rs13107325’s uniquely strong association with cortico-hippocampal connectivity (see previous section) is consistent with the long literature relating hippocampal size, morphology, function and connectivity to schizophrenia^90^. rs13135092 has 477 reported associations in the GWAS Catalog, including with a wide variety of brain imaging measures.

The fifth-most significant variant, rs12146713 (chr12:106,476,805), is an intronic variant in *NUAK1*. NUAK1 dose-dependently regulates axon elongation and branching via effects on mitochondrial metabolism and trafficking^91–93^. NUAK1 is a candidate gene for autism spectrum disorder (ASD), attention-deficit/hyperactivity disorder (ADHD) and intellectual disability. In mice, NUAK1 haploinsufficiency impairs cortical development and has broad-spectrum effects on cognition^92^. The GWAS Catalog reports 73 associations for rs12146713, all brain structure-related.

The sixth-most significant variant, rs35050623 (chr2:37,063,240), is an intergenic variant with *STRN* as the nearest protein-coding gene. STRN (striatin) is a calmodulin-binding protein enriched in dendritic spines^94,95^. In cultured striatal neurons, *STRN* knockdown led to increased dendritic arborization and increased density of stubby spines (a type of dendritic spine with no neck), indicating a role in regulating striatal neurodevelopment^96^. rs35050623 has one GWAS Catalog association, with brain shape.

The eighth-most significant variant, rs4799450 (chr18:35,028,901), is an intronic variant in *CELF4*. CELF4 is an RNA binding protein and translational regulator of prenatal neocortical subplate layer-based synaptic development^97^. CELF4 is involved in differentiation and excitation of neurons, corticothalamic development, synaptic transmission, and neuroplasticity and is an important regulator of mRNA stability and translational availability^98^. Deficiency can lead to dysfunctional neuronal excitation and impaired synaptic transmission^99^. CELF4 dysfunction has been linked to a variety of neuropsychiatric disorders including autism, bipolar disorder, schizophrenia, and epilepsy^98^. rs4799450 has one GWAS Catalog association, with white matter microstructure.

The ninth-most significant variant, rs199790004 (chr8:26,427,757), is an intronic variant in *DPYSL2*. DPYSL2 is a microtubule-stabilizing protein that plays a role in the regulation of axonal outgrowth, dendritic development, synapse elongation and vesicle trafficking during neurodevelopment^100^ and is a key regulator of neural stem cell differentiation^101^. *DPYSL2* is a known schizophrenia (SCZ) risk gene and its expression is altered in schizophrenia patient brains^102–104^, possibly as a downstream consequence of disrupted mTOR signaling^103,104^.

The tenth-most significant variant, rs35124509 (chr3:89,521,693), is a missense variant in *EPHA3*, the sole protein-coding gene for over 1 megabase in either direction. Knockdown of EPHA3 disrupts pathfinding of axons through the corpus callosum^105^. EPHA3 activation reduces neurite outgrowth and, more specifically, induces growth cone collapse^106^. EPHA3 also affects higher-level structural connectivity patterns, regulating excitation-inhibition balance and GABAergic interneuron synaptic density^107,108^. rs35124509 has 16 GWAS Catalog associations, all with brain imaging measurements.

The eleventh-most significant variant, rs150346963 (chr7:117,625,599), is an intergenic variant located ~100 kilobases upstream of *CTTNBP2*. CTTNBP2 (cortactin binding protein 2) is a cytoskeletal protein predominantly expressed in neurons and controls synapse and dendritic spine formation and maintenance by interacting with cortactin, which binds to and stabilizes actin filaments^109–111^. *CTTNBP2* is a candidate gene for autism spectrum disorder^109–113^ and mice with autism spectrum disorder-linked mutations in *CTTNBP2* knocked in have impaired synaptic function^110^. Meanwhile, *CTTNBP2* knockout mice have reduced brain zinc levels – a risk factor for autism – and altered synaptic protein targeting, while zinc supplementation rescues synaptic retention of CTTNBP2 and improves social behaviors in these mice^112,113^. rs150346963 has one GWAS Catalog association, with major depressive disorder.

The twelfth-most significant variant, rs118087478 (chr17:44,051,589), is an intronic and 5’ UTR variant in *MAPT*. MAPT (tau) is best known for its central role in neurodegenerative disorders, but it is also involved in neurodevelopment, where it is highly expressed^114^ and regulates the assembly and stability of microtubules and their linkage to the plasma membrane^115^. Microdeletions in the *MAPT* genetic region (at chromosome 17q21.3) have been linked to intellectual disabilities^116^, which is proposed to be the result of altered tau dosage^114^, and *Mapt* knockout leads to a reduction in autistic behaviors in mouse models of autism^117^. rs118087478 has one GWAS Catalog association, with brain volume.

The 14^th^-most significant variant, rs79814107 (chr14:59,637,503), is an intronic variant in *DAAM1*. DAAM1 (disheveled associated activator of morphogenesis 1) is a scaffolding protein that acts downstream of Wnt signaling and controls cell polarity and movement by regulating actin cytoskeleton organization^118,119^. In mice, deletion of a neural-specific microexon in *Daam1* led to memory defects, reduced long-term potentiation and a decrease in the number of dendritic spines^120^. rs79814107 has been previously associated with brain volume and white matter integrity according to the GWAS Catalog.

The 15^th^-most significant variant, rs34844662 (chr13:109,687,510) is an intronic variant in *MYO16*. MYO16 is an unconventional myosin that is involved with cytoskeleton remodeling and is predominantly expressed in the central nervous system^121^. Within neurons, MYO16 interacts with the WAVE regulatory complex which modulates dendritic spine morphology by regulating actin polymerization, particularly in Purkinje cells, and synaptic organization^121,122^. *MYO16* is a candidate gene for bipolar II disorder^123^, schizophrenia^124^, and autism^125^.

The 16^th^-most significant variant, rs6558407 (chr8:144,995,494), is a missense variant in *PLEC*. PLEC (plectin) is a ‘cytolinker’ protein that cross-links cytoskeletal elements with each other, including intermediate filaments, actin filaments, microtubules, and components of the cell and nuclear membranes^126^. Plectin is highly expressed in the central nervous system, especially at the interfaces between glia and pial cells and between glia and endothelial cells, and is thought to be important to blood-brain barrier and pial surface integrity^127^. Plectin has been linked to epilepsy and Alexander disease, a rare demyelinating disease^128^. Mice genetically deficient in the plectin isoform P1c had poorer learning capabilities and long-term memory than wild-type littermates^129^.

The 17^th^-most significant variant, rs59154421 (chr2:162,813,034), is an intronic variant in *SLC4A10*. SLC4A10 is a transporter in the plasma membrane that is predominantly expressed in neurons. It helps regulate neuronal pH by taking in bicarbonate, driven by a sodium gradient across the membrane, which helps remove hydrogen ions^130,131^. Case studies have shown that autosomal recessive loss of function of *SLC4A10* leads to intellectual disability, microcephaly and lateral ventricle abnormalities^130^. *Slc4a10* knockout mice have smaller brain ventricles and behavioral abnormalities, and SLC4A10 modulates GABA (but not glutamate) release in mouse brain^130^. *SLC4A10* is a candidate gene for autism spectrum disorder^132^ and epilepsy^133^.

The 19^th^-most significant variant, rs13228652 (chr7:83,761,158), is an intronic variant in *SEMA3A*. Semaphorins are a family of membrane proteins that orchestrate axon guidance and induce growth cone collapse (leading to their alternate name, collapsins)^134,135^. SEMA3A (semaphorin 3A, also known as collapsin-1 or collapsin) was the first-discovered semaphorin^134^ and acts as an axonal chemorepellent (i.e., it repels growth cones) as well as inducing growth cone collapse. In addition to this primary role, SEMA3A also has an important secondary role in inhibiting myelin regeneration in multiple sclerosis, at least in part by inhibiting oligodendrocyte precursor cell differentiation and recruitment to demyelinated lesions^136–139^.

The 22^nd^-most significant variant, rs975360 (chr7:81,411,028) is ~10 kilobases upstream of the transcription start site of *HGF*. HGF (hepatocyte growth factor) is a pleiotropic cytokine that provides trophic (growth, differentiation and survival) support to neurons and is involved in promoting cell movement, axon growth and dendritic morphology^140,141^. HGF is expressed in the cortex and hippocampus during neurodevelopment where it regulates thalamocortical axon growth^142^. HGF’s cognate receptor, the tyrosine kinase *MET*, is a high-confidence autism risk gene, and MET protein levels are lower in the brains of people with autism compared to healthy controls^143^. A mouse model of decreased HGF and MET expression showed deficiencies in interneuronal migration during neurodevelopment^144^.

The 28^th^-most significant variant, rs5788199 (chr10:118,649,704), is an intronic variant in two overlapping genes, *SHTN1* and *ENO4*. SHTN1 is necessary for neuronal polarization, the process by which one of the neurites from a developing neuron ultimately becomes the axon and the rest become dendrites. SHTN1 builds up asymmetrically in the neurite that ultimately becomes the axon, and disrupting this asymmetrical accumulation either by overexpression or knockdown disrupts neuronal polarization^145^. *Shtn1* knockout mice exhibited thinning of multiple white matter tracts—the corpus callosum, anterior commissure and hippocampal commissure—as well as absence of the septum^146^.

### Structural connectivity is highly polygenic and exhibits signatures of negative selection

**Fig. 5:**
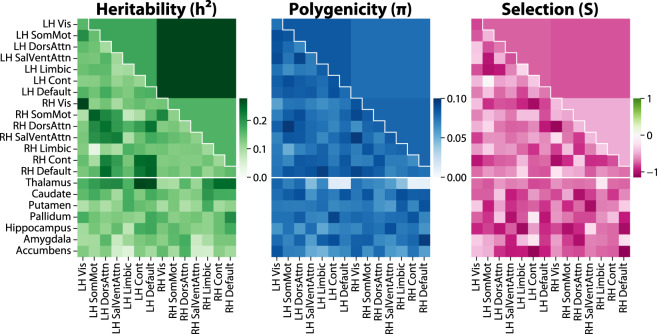
Global properties of the genetic architecture of structural connectivity. LH left-hemisphere, RH right-hemisphere, Vis visual, SomMot somatomotor, DorsAttn dorsal attention, SalVentAttn salience/ventral attention, Limbic limbic, Cont control, Default default mode.

We next inferred global properties of the genetic architecture of each structural connectivity measure by applying the recently developed SBayesS method^147^ (Fig. 1, Fig. 5).

The proportion of heritability (*h*^2^) collectively explained by the 9,423,516 common variants included in the GWAS was relatively modest, ranging from 3.3 to 27.7% (median 13.9%) across the 206 GWAS. As expected, the most heritable measures were also the most replicable between the initial and the follow-up scans (Pearson correlation between heritability and replicability across measures = 0.77), indicating greater stability of these measures over time. Estimates of *h*^2^ from LD score regression^148^ were lower, ranging from 1.0 to 18.0% (median 9.1%), but displayed a similar pattern across the 206 measures (Supplementary Fig. 2). The discrepancy in *h*^2^ between the two methods may be attributable to the different assumptions about genetic architecture: in particular, SBayesS assumes a sparse genetic architecture, while LD score regression does not make such an assumption. Heritability was higher for inter-hemisphere connectivity (*h*^*2*^ = 27.7%) than either left intra-hemisphere (*h*^*2*^ = 18.0%) or right intra-hemisphere (*h*^*2*^ = 16.3%) connectivity.

Polygenicity (*π*) was quite high, with between 0.8% and 9.6% (median 7.5%) of common variants surveyed estimated to have non-zero effects on each of the 206 measures. Strikingly, corticothalamic connectivity measures involving the default-mode and control networks were among the least polygenic (*π* = 0.8–1.2%, median 1.0%) despite being among the most heritable (*h*^*2*^ = 24.2–27.5%, median 25.7%). The combination of low polygenicity and high heritability suggests that these corticothalamic connections are controlled by more specific gene sets than other types of connections. This observation is consistent with our identification of variants with disproportionate associations with corticothalamic connectivity overall, near *CCDC88C*, *GMNC*, *NUAK1*, and *INPP5D* (Fig. 4).

SBayes also estimates a selection parameter (*S*), where -1 indicates strong negative selection, 0 indicates no selection, and +1 indicates strong positive selection. The degree to which a trait is under selection can be inferred from the relationship between minor allele frequency and GWAS effect size. For instance, if a trait is under strong negative selection, variants with large effects on the trait will tend to be rare, since individuals who carry these variants will have lower evolutionary fitness and be less likely to pass on their genes. 194 of the 206 measures were estimated to be under negative selection (95% confidence interval of *S* entirely below 0), indicating significantly larger effect sizes among lower-frequency variants. All 206 measures had point estimates of *S* below 0, ranging from –1.13 to –0.03 (median –0.70).

Overall, the high polygenicity of these measures indicates that a substantial fraction of the genome is involved in genetically determining structural connectivity, and remains to be discovered by future GWAS. Meanwhile, the fact that these measures appear to be under negative selection suggests that variants affecting structural connectivity tend to have negative consequences on evolutionary fitness.

### Structural connectivity is genetically correlated with a range of psychiatric and cognitive traits

We computed genetic correlations (r_g_) between our 206 GWAS and 15 well-powered GWAS for neurological, psychiatric, neurodevelopmental, and cognitive traits, using the genetic covariance analyzer (GNOVA) method^149^. We chose GNOVA due to its improved correction for sample overlap relative to cross-trait LD score regression^150^, another commonly used genetic correlation method. After Bonferroni correction across the 206 × 15 genetic correlations tested, we observed 18 significant genetic correlations (Supplementary Fig. 3). 6 traits had at least one significant genetic correlation (Fig. 1): bipolar disorder, ADHD, insomnia, Alzheimer’s disease, educational attainment, and reaction time. These significant genetic correlations were positive for ADHD (r_g_ = 0.19–0.24), insomnia (r_g_ = 0.16), and Alzheimer’s disease (r_g_ = 0.23), indicating that the genetic variants associated with increased connectivity tended to be the same genetic variants associated with increased risk for these conditions, when looking across the whole genome rather than only at genome-wide significant variants. Conversely, the significant genetic correlations were negative for bipolar disorder (r_g_ = –0.17), educational attainment (r_g_ = –0.22 to –0.11), and reaction time (r_g_ = –0.18 to –0.13), indicating that the genetic variants associated with increased connectivity tended to be the same genetic variants associated with reduced risk for bipolar disorder, lower educational attainment, and – somewhat paradoxically given the educational attainment result – better reaction time. On the whole, these genetic correlations support the relevance of genetic factors influencing structural connectivity to a range of neuropsychiatric and cognitive outcomes.

We also computed phenotypic and genetic correlations between the three hemisphere-level measures and 432 diffusion MRI measures based on TBSS and neurite orientation dispersion and density imaging (NODDI) in the UK Biobank (Supplementary Data 3), leveraging prior GWAS of these measures^18^. Cross-hemisphere connectivity had Bonferroni-significant phenotypic and genetic correlations with 401 and 197 of the 432 measures, respectively; left-hemisphere connectivity with 381 and 155; and right-hemisphere connectivity with 375 and 100. Phenotypic correlations ranged from -0.60 (between cross-hemisphere connectivity and “Mean OD [orientation dispersion] in cerebral peduncle on FA [fractional anisotropy] skeleton (left)”) to 0.53 (between cross-hemisphere connectivity and “Mean FA in cerebral peduncle on FA skeleton (left)”). Genetic correlations ranged from -0.59 (between cross-hemisphere connectivity and “Mean MO [mean orientation] in body of corpus callosum on FA skeleton”) to 0.53 (between cross-hemisphere connectivity and “Mean ISOVF [isotropic volume fraction] in body of corpus callosum on FA skeleton”). On the whole, this analysis indicates that structural connectivity is phenotypically and genetically related, but nonetheless substantially distinct, from more widely used diffusion MRI measures.

### Structural connectivity heritability is enriched in regions with increased chromatin accessibility in oligodendrocytes

We applied partitioned linkage disequilibrium score regression^151^ to investigate whether heritability for each structural connectivity measure was enriched in regions with increased chromatin accessibility in each of 6 major brain cell types—astrocytes, excitatory neurons, inhibitory neurons, microglia, oligodendrocytes, and oligodendrocyte precursor cells (OPCs)—measured by single-nucleus chromatin accessibility and messenger RNA expression sequencing (SNARE-seq2) of postmortem adult human motor cortex^152^. After Bonferroni correction across the 206 × 6 cell-type enrichments tested, we found significant enrichments for 7 structural connectivity measures across four cell types (Fig. 1, Supplementary Fig. 4): oligodendrocytes with connectivity within the right-hemisphere default-mode network (67-fold enrichment, 95% confidence interval 32–101, *p* = 3 × 10^–5^), microglia with 3 measures, most significantly left-hemisphere dorsal attention to salience/ventral attention connectivity (52-fold [29-76], *p* = 4 × 10^–7^), inhibitory neurons with right-hemisphere dorsal attention to right-hemisphere limbic connectivity (49-fold [28-69], *p* = 5 × 10^–6^), and astrocytes with 2 measures, most significantly left- to right-hemisphere visual network connectivity (48-fold [27–69], *p* = 5 × 10^–6^). There were many sub-significant enrichments as well, particularly for astrocytes and oligodendrocytes. Given that oligodendrocytes are the cell type responsible for myelination and one of the primary functions of oligodendrocytes is to myelinate white matter fibers^153^, the observed oligodendrocyte heritability enrichment suggests that genetic control of structural connectivity may be partially mediated by effects on myelination.

We performed the same cell-type enrichment analysis across 12 cell types from the developing human cortex, using published single-cell assay for transposase-accessible chromatin with high-throughput sequencing (ATAC-seq) data^154^. After Bonferroni correction across the 206 × 12 cell-type enrichments tested, we found significantly enriched heritability in four cell types, all non-neuronal (Supplementary Fig. 5). These cell types were: astrocytes and oligodendrocyte progenitor cells (for right-hemisphere visual network to pallidum connectivity: 70-fold enrichment, 95% confidence interval 34-106, *p* = 2 × 10^–5^), endothelial/mural cells (for 6 structural connectivity measures, with enrichments between 98- and 125-fold), microglia (19 measures, 65- to 126-fold), and radial glial cells (18 measures, 35- to 82-fold), stem cells that are progenitors of both neurons and specific glia, namely astrocytes and oligodendrocytes. (We also found significantly disenriched heritability in insular neurons.) These heritability enrichments for multiple fetal cell types suggest that structural connectivity is partially mediated by effects on early brain development, particularly in non-neurons.

## Discussion

In this study, we describe the genetic architecture of white-matter structural connectivity via genome-wide association studies of 206 tractography-derived measures across 26,333 UK Biobank participants. We discover 30 loci associated with structural connectivity after rigorous correction for multiple statistical tests performed (with 126 loci at nominal genome-wide significance). These loci pinpoint neurodevelopmental genes with influences on myelination (*SEMA3A*), neurite elongation and guidance (*NUAK1*, *STRN*, *DPYSL2*, *EPHA3*, *SEMA3A*, *HGF*, *SHTN1*), neural cell proliferation and differentiation (*GMNC*, *CELF4*, *HGF*), neuronal migration (*CCDC88C*), cytoskeletal organization (*CTTNBP2*, *MAPT*, *DAAM1*, *MYO16*, *PLEC*), and brain metal transport (*SLC39A8*). In addition to these biological processes, we observe enriched heritability for structural connectivity in regions with increased chromatin accessibility in oligodendrocytes, microglia, inhibitory neurons, astrocytes, and multiple fetal cell types, supporting a role for myelination and early brain development in the genetics of structural connectivity. Our results extend the rich literature of MRI GWAS measuring other aspects of brain structure and function.

We show, for the first time, that structural connectivity is highly polygenic: we estimate that for the participants in our cohort, the average connectivity measure is influenced by 9.1% of all common (>1% frequency) variants in the genome. This suggests that our 30 loci represent only a small fraction of genetic influences on the structural connectome; much larger sample sizes, possibly in the tens of millions, may be needed to discover the rest^155^. This high polygenicity is accompanied by evidence of negative selection: higher-frequency variants tend to have smaller effects on structural connectivity. Together, these results imply a genetic architecture whereby large numbers of disproportionately common variants each exert small effects on structural connectivity, while larger-effect variants tend to be flushed out of the population due to their deleteriousness. Both high polygenicity^22,155^ and strong negative selection^22^ appear to be characteristic of brain MRI measures in general, rather than being unique properties of structural connectivity in particular. On the other hand, the 18 UK Biobank traits analyzed in the SBayesS paper^147^ tend to be less polygenic than our structural connectivity measures, despite having similar evidence of negative selection.

Far from providing a one-to-one map between specific genetic variants and specific regional connectivities, our results indicate widespread pleiotropy of structural connectivity-associated variants. Our 30 lead variants are each associated with a median of 7.5 of the 206 measures, and many are also associated with other aspects of brain structure. The spatial associations of these variants fall into four broad patterns: three variants are disproportionately associated with interhemispheric and corticothalamic connectivity, one with corticothalamic connectivity alone, and eight with interhemispheric alone, while the remaining 18 have spatially diffuse associations. Corticothalamic connectivity measures involving the default-mode and control networks are among the least polygenic despite being among the most heritable, providing further evidence in support of corticothalamic connections being controlled by more specific gene sets than other types of connections. Corticothalamic structural connectivity may have outsized importance relative to other types of structural connections, as it underpins cortico-thalamo-cortical loops, modular units of brain organization involved in consciousness and perception^156^.

Genetic correlation analyses indicate shared genetic influences between structural connectivity and a range of neuropsychiatric and cognitive traits. The directionality of these associations is somewhat counterintuitive: variants associated with increased structural connectivity tended to be associated with increased risk of ADHD, insomnia, and Alzheimer’s disease, decreased risk of bipolar disorder, lower educational attainment, and better reaction time. While cognitive decline has been associated with reduced structural connectivity^56,157^, and cognitive resilience with increased connectivity^158^, in mild cognitive impairment, Alzheimer’s disease and/or aging, the relationship between structural connectivity and cognition in healthy individuals after accounting for age has been less well-studied. Note that the Alzheimer’s disease GWAS we used included proxy cases for Alzheimer’s disease and related dementias, which increases power but can also lead to bias in genetic epidemiological analysis^159^.

In parallel to ours, another study also examined the genetic architecture of white-matter structural connectivity in the UK Biobank^160^. Unlike our study, the authors used a multivariate GWAS technique, MOSTest^161^, to boost power by leveraging the fact that GWAS variants associated with structural connectivity tend to be associated with connectivity changes in multiple parts of the brain. While focused less on the mechanisms underlying individual causal gene candidates, the authors nonetheless show a global enrichment of their GWAS associations for many of the same neurodevelopmental processes as we report for our individual causal gene candidates, including neurogenesis, neural differentiation, neural migration, neural projection guidance, and axon development. As in our study, the authors report pervasive shared genetics between structural connectivity and neuropsychiatric disease.

This study has several important limitations. First, the core limitation of our study is the use of tractography to infer structural connectivity. The term “structural connectivity” and the assumption that we can infer connectivity at all from diffusion MRI-based methods is inherently limited; this is true of all cases of using MRI derived metrics to infer neurobiological details. Unlike ex vivo methods like histological tract-tracing, tractography is not a direct measure of structural connectivity. The measurements it is based on relate to the diffusion of water molecules in tissue, with tissue structure or organization influencing the diffusivity. This is far removed from true structural connectivity (at the level of axons), and thus we are building at best an estimated model of large-scale trends of connectivity. Tractography has trouble resolving small or intermingled axon bundles, and it is vulnerable to false positive connections^162,163^. Moreover, tractography is ill-suited to measuring the short-range and unmyelinated connections typical of gray matter. Different tractography algorithms can also have differences based on tractography models and parameters^164^, which can vary by tract^165^. Our approach attempted to use current best practices to minimize the generation of spurious tracts and assess reliability. As diffusion algorithms and imaging techniques improve, more reliable and interpretable metrics will likely be derived. This could lead to the identification of metrics that relate more specifically to different types of biological variation (e.g. myelination versus packing density) and thereby allow the identification of genetic variation specifically related to different aspects of structural variation. As such, while we were able to identify relationships between these large scale models of structural connectivity and genetics, these results should be interpreted within the limitations of current diffusion imaging approaches. These limitations should be weighed against the unique opportunity and scalability afforded by diffusion MRI-based tractography to uncover genetic influences on the physical wiring of the human brain.

Second, our results were derived from MRI scans of participants aged 40–70 (median 55), and may not generalize to participants of other age groups. In particular, age-related structural connectivity changes are associated with cognitive decline^166^, which may be related to our unexpected observation of negative genetic correlations between structural connectivity and cognitive measures.

Third, the 206 structural connectivity measures we defined are inherently somewhat arbitrary, and other measures might uncover complementary biological insights. For instance, we chose to define cortical measures at the level of Yeo 7 networks, which are defined based on functional, rather than structural, connectivity. While we feel this choice has substantial advantages in terms of interpretability, defining cortical networks in a data-driven way, based on the tractography data itself, is an alternative possibility. Other possibilities include considering subcortical structures in a hemisphere-specific manner (e.g. considering left default mode to left amygdala connections separately from left default mode to right amygdala connections), including subcortical-to-subcortical connections, and incorporating measures of overall network topology like network efficiency^56,59^ and rich-club organization^167,168^. Exemplifying the kinds of insights that can be gained from genetic analyses of these more complex measures, a recent twin study found that heritability of structural connectivity was concentrated in rich-club connections, i.e. those interlinking network hubs^169^.

In sum, our results indicate pervasive genetic influences on structural connectivity within the human brain and support their relevance to brain function in health and disease. Future studies using more sophisticated imaging techniques will be necessary to extend these results to gray-matter structural connectivity and develop a truly comprehensive map of genetic influences on the human structural connectome.

## Methods

### Cohort selection and MRI quality control

In total, 41,489 participants from the UK Biobank were listed as having both T1 (Data-Field 20263, “T1 surface model files and additional structural segmentations”) and diffusion-weighted (Data-Field 20250, “Multiband diffusion brain images - NIFTI”) MRI scans available at baseline. However, of these baseline scans, 1456 lacked diffusion-weighted MRI scans corrected for eddy currents^170^, head motion, outlier slices, and gradient distortion (data_ud.nii.gz from Data-Field 20250); a further 8725 lacked eddy-corrected bvec files (data.eddy_rotated_bvecs from Data-Field 20250); and a further 3 lacked FreeSurfer parcellation images (aseg.mgz from Data-Field 20263). This left 31,309 participants.

We next considered four quality control metrics provided by the UK Biobank: T1 inverse signal-to-noise ratio (“Inverted signal-to-noise ratio in T1”, Data-Field 25734), T1 inverse contrast-to-noise-ratio (“Inverted contrast-to-noise ratio in T1”, Data-Field 25735), number of diffusion MRI outlier slices (“Number of dMRI outlier slices detected and corrected”, Data-Field 25746), and left-to-right head motion as measured by eddy in the diffusion MRI (“Standard deviation of apparent translation in the Y axis as measured by eddy”, Data-Field 25922). All four quality control metrics were defined so that higher values are worse. Noting that the distributions of all four metrics were right-skewed to varying degrees, with a tail of poor-quality scans, we excluded scans where any of the four metrics were more than 3 standard deviations above the mean (Supplementary Fig. 6). 1113 participants had baseline scans excluded, leaving 30,196 participants remaining.

In total, 26,445 of these participants were of European genetic ancestry (according to Pan-UK Biobank, Return 2442) and also had genotyping data deemed suitable for genetic analyses: genotypes available, no mismatch between genetic sex (“Genetic sex”, Data-Field 22001) and self-reported sex (“Sex”, Data-Field 31), no sex chromosome aneuploidy (“Sex chromosome aneuploidy”, Data-Field 22019), and not flagged as “Outliers for heterozygosity or missing rate” (Data-Field 22027). An additional 668 participants were of non-European genetic ancestry (Central/South Asian, African, East Asian, Middle Eastern or Admixed American) and had genotyping data deemed suitable for genetic analyses.

Thus, we ran our tractography pipeline (see next section) on 27,113 baseline scans, of which 26,998 ran successfully: 26,333 of European genetic ancestry and 665 of non-European genetic ancestry. We used the 26,333 European baseline scans for the main GWAS, and the 665 non-European baseline scans for the replication GWAS. To evaluate replicability of the tractography measures themselves, we used a subset of 2400 participants of European genetic ancestry who also had replicate scans that passed all the above-mentioned filters and ran successfully.

### Tractography pipeline

Our tractography pipeline, tractify (https://github.com/TIGRLab/tractify), performs probabilistic tractography using version 3.0.3 of the MRtrix3 diffusion MRI software package^69^. Certain steps also use version 6.0.5 of the Functional Magnetic Resonance Imaging of the Brain Software Library (FSL)^171^ and version 7.1.1 of the FreeSurfer software package^172^.

The pipeline takes the following subject-specific inputs:

1. A brain-extracted, bias-field-corrected T1 scan (brain.mgz from Data-Field 20263), the processing of which is described in detail in the UK Biobank Brain Imaging Documentation.
2. A diffusion-weighted MRI scan corrected for eddy currents, head motion, outlier slices, and gradient distortion (data_ud.nii.gz from Data-Field 20250), the processing of which is also described in the UK Biobank Brain Imaging Documentation.
3. A bval file (bvals from Data-Field 20250).
4. An eddy-corrected bvec file (data.eddy_rotated_bvecs from Data-Field 20250).
5. A FreeSurfer parcellation of the T1 image (aseg.mgz from Data-Field 20263). and the following subject-independent inputs:
6. The 2-mm MNI152 standard-space structural template image, MNI152_T1_2mm_brain.nii.gz.
7. The atlas: we concatenated the 200-parcel Schaefer cortical atlas^73^ mapped to the “Yeo 7” networks^67^, available from https://github.com/ThomasYeoLab/CBIG/tree/master/stable_projects/brain_parcellation/Schaefer2018_LocalGlobal/Parcellations/MNI/Schaefer2018_200Parcels_7Networks_order_FSLMNI152_2mm.nii.gz, with 14 subcortical parcels from the Harvard-Oxford atlas (left thalamus, left caudate, left putamen, left pallidum, left hippocampus, left amygdala, left accumbens, right thalamus, right caudate, right putamen, right pallidum, right hippocampus, right amygdala, right accumbens), for a total of 214 parcels. Voxels present in both a Schaefer parcel and a subcortical Harvard-Oxford parcel were assigned to their Schaefer rather than their Harvard-Oxford parcel. This atlas is available as Supplementary Data 4.

The pipeline consists of the following steps for each subject:

#### Part 1: Extract the mean b = 0 image

1. Perform bias field correction on the diffusion MRI scan (input #2) via the N4 algorithm^173^, using MRtrix3’s dwibiascorrect ants command.
2. Extract the mean b = 0 image from the bias-field-corrected scan (output at step 1). First, use MRtrix3’s dwiextract -bzero command, supplying the bvals and eddy-corrected bvecs (inputs #3 and #4) via the -fslgrad flag. Then, average across volumes using MRtrix3’s mrmath -axis 3 mean command.
3. Skullstrip the mean b = 0 image (output at step 2) using FSL’s bet command with the default fractional intensity threshold (-f) of 0.5. Enable robust brain center estimation via the -R flag. Specify the -m flag to generate a binary brain mask in addition to the skullstripped mean b = 0 image.

#### Part 2: Nonlinearly register the atlas to diffusion space (via T1 space, to improve registration quality)

4. Convert the T1 scan (input #1) from MGZ to NIfTI format using MRtrix3’s mrconvert command.
5. Linearly register the T1 scan from native space to diffusion space using FSL’s flirt command^174,175^. Provide the T1 scan in NIfTI-format (output at step 4) via the -in flag and the skullstripped mean b = 0 image (output at step 3) via the -ref flag. Use 6 degrees of freedom (-dof 6) for the registration. This yields two outputs: the diffusion-space T1 scan (-out), and the T1-to-diffusion transformation matrix (-omat).
6. Linearly register the T1-weighted image from native space to the MNI152 template using FSL’s flirt command. Provide the T1 scan in NIfTI format (output at step 4) via the -in flag, the MNI152 template (input #6) via the -ref flag, and the location to save the T1-to-MNI152 transformation matrix via the -omat flag. Use the default 12 degrees of freedom.
7. Nonlinearly register the T1-weighted image from native space to the MNI152 template by leveraging the linear registration from step 6, using FSL’s fnirt command. Provide the T1 scan in NIfTI format (output at step 4) via the --in flag, the T1-to-MNI152 transformation matrix (output at step 6) via the --aff flag, the MNI152 template (input #6) via the --ref flag, and the location to save the warping transformation via the --cout flag. Specify --config=T1_2_MNI152_2mm.cnf to use FSL’s internal configuration file T1_2_MNI152_2mm.cnf, as recommended by the creators of FSL when registering T1-weighted images to the MNI152 template.
8. Compute the MNI152-to-T1 warping transformation by inverting the T1-to-MNI152 warping transformation, using FSL’s invwarp command. Provide the T1-to-MNI152 warping transformation (output at step 7) via the -w flag, the T1 scan in NIfTI format (output at step 4) via the -r flag, and the location to save the MNI152-to-T1 warping transformation via the -o flag.
9. Register the atlas from MNI152 space to diffusion space using FSL’s applywarp command, by applying the non-linear MNI152-to-T1 warping transformation from step 8, followed by the linear T1-to-diffusion transformation from step 5. Provide the atlas (input #7) via the -i flag, the the skullstripped mean b = 0 image (output at step 3) via the -r flag, the MNI152-to-T1 warping transformation (output at step 8) via the -w flag, the T1-to-diffusion transformation matrix (output at step 5) via the --postmat flag, and the location to save the diffusion-space atlas via the -o flag. Specify nearest-neighbors interpolation via the --interp = nn flag, and specify the --ref flag to treat the warp field as relative.

#### Part 3: Generate a five-tissue-type image and extract the gray-white matter interface

10. Convert the Freesurfer parcellation image (input #5) from MGZ to NIfTI format using MRtrix3’s mrconvert command.
11. Register the Freesurfer parcellation image from T1 space to diffusion space using FSL’s flirt command. Provide the parcellation image in NIfTI format (output at step 10) via the -in flag, the T1-to-diffusion transformation matrix (output at step 5) via the -applyxfm -init flags, and the location to save the diffusion-space parcellation image via the -out flag. (We also provide the diffusion-space T1 scan, output at step 5, via the -ref flag, but only so that flirt knows which voxel and image dimensions to use for the output.) Use nearest-neighbor interpolation (-interp nearestneighbour). Use the default 12 degrees of freedom.
12. Generate a five-tissue-type image from the diffusion-space Freesurfer parcellation image (output at step 11) using MRtrix3’s 5ttgen freesurfer command. Specify the -nocrop flag to keep the same dimensions as the input image.
13. Extract the gray-white matter interface from the five-tissue-type image (output at step 12) using MRtrix3’s 5tt2gmwmi command.

#### Part 4: Perform probabilistic tractography

14. Generate multi-shell multi-tissue response functions using MRtrix3’s dwi2response msmt_5tt command. Provide the diffusion MRI scan (input #2), the five-tissue-type image (output at step 12), the bvals and eddy-corrected bvecs (inputs #3 and #4) via the -fslgrad flag, and the binary brain mask (output at step 3) via the -mask flag. This yields three outputs: response functions for the white matter, gray matter and cerebrospinal fluid.
15. Estimate a fiber orientation distribution (FOD) via multi-shell multi-tissue constrained spherical deconvolution (MSMT-CSD)^70^ using MRtrix3’s dwi2fod msmt_csd command. Provide all of the same inputs as step 14 as well as all of the outputs of step 14. This yields three outputs: orientation distribution function (ODF) images for the white matter, gray matter and cerebrospinal fluid. We will only use the white-matter ODF.
16. Perform probabilistic tractography using MRtrix3’s tckgen command. Provide the white-matter ODF (output at step 15). Use anatomically-constrained tractography (ACT)^176^ by providing the five-tissue-type image (output at step 12) via the -act flag. Seed streamlines from the gray-white matter interface (output at step 13) by providing it via the -seed_gmwmi flag. Use the Second-order Integration over Fiber Orientation Distributions (iFOD2) algorithm^71^ by specifying -algorithm iFOD2. Select 1 million streamlines (-select 1000000).
17. Weight each streamline so that the density of reconstructed connections better reflects the density of the underlying white matter fibers via the SIFT2 algorithm^72^, as implemented in MRtrix3’s tcksift2 command. Provide the streamlines (output at step 16) and the white-matter ODF (output at step 15).
18. Generate a connectome matrix using MRtrix3’s tck2connectome command, denoting the weighted number of streamlines connecting each pair of parcels in the atlas (in our case, this matrix has dimension 214 × 214). Provide the streamlines (output at step 16) and the diffusion-space atlas (output at step 9). Also provide the weight for each streamline (output at step 17) via the -tck_weights_in flag. Perform a radial search from each streamline endpoint to locate the nearest parcel by specifying the -assignment_radial_search flag^177^. Require the matrix to be symmetric and have a diagonal of 0 via the -symmetric and -zero_diagonal flags. Weighted the entries in the connectome matrix by both the length of the streamline (-scale_length), to account for the bias of probabilistic tractography towards shorter connections, and the inverse of the product of the two parcel volumes (-scale_invnodevol)^178^, to correct for the variability in the size of parcels that would otherwise result in higher connectivity to/from larger parcels solely due to their size.

### Phenotype definitions

We defined a total of 3 hemisphere-level connectivity measures (left intra-hemisphere, right intra-hemisphere, inter-hemisphere). Specifically, we averaged the entries of the connectome matrix across all pairs of parcels in the left hemisphere to obtain the overall connectivity within the left hemisphere, and similarly for the right hemisphere. We quantified overall inter-hemispheric connectivity by averaging the entries connecting any parcel in the left hemisphere to any parcel in the right hemisphere.

We defined a total of 105 network-level connectivity measures. Each parcel in the Schaefer atlas has previously been mapped to one of 7 large-scale brain networks, commonly known as the “Yeo 7” networks, which were derived in a data-driven manner based on functional connectivity^67^: visual (“Vis”), somatomotor (“SomMot”), dorsal attention (“DorsAttn”), salience/ventral attention (“SalVentAttn”), limbic, control (“Cont”), and default mode (“Default”). Because each network is present in both hemispheres, there are 14 hemisphere-specific networks, denoted e.g. “LH Vis” for the left-hemisphere visual network and “RH Cont” for the right-hemisphere control network. For each network, we averaged the entries of the connectome matrix across all pairs of parcels in that network to define 14 within-network connectivity measures. Also, for each pair of networks, we averaged the entries of the connectome matrix connecting any parcel in the first network to any parcel in the second network, to define 91 between-network connectivity measures.

Finally, we defined a total of 98 cortical-to-subcortical connectivity measures, between each of the 14 hemisphere-specific Yeo 7 networks and each of the 7 subcortical structures (thalamus, caudate, putamen, pallidum, hippocampus, amygdala, and accumbens).

### Genome-wide association studies

We performed genome-wide association studies for the 206 connectivity measures by linearly regressing each measure on each of 9,423,516 single-nucleotide genetic variants (see below) using version 3.2.9 of the regenie genetic analysis toolkit (https://rgcgithub.github.io/regenie)^74^. regenie accounts for sample relatedness and population structure by computing a polygenic risk score (PRS) for the phenotype being associated (step 1), then including this PRS as a covariate when performing the actual association testing (step 2). regenie uses a leave-one-chromosome-out scheme to construct the PRS (e.g. when performing association tests on chromosome 19, a PRS derived from all chromosomes except chromosome 19 is used as a covariate).

For step 1, we used a quality-controlled subset of the UK Biobank’s unimputed genotype data, namely the 596,935 variants on the autosomes and the non-pseudoautosomal regions of the X chromosome with minor allele frequency >1%, <10% missingness and Hardy-Weinberg equilibrium *p* > 1 × 10^–15^ (with mid-P correction) across all 425,630 UK Biobank participants of European genetic ancestry (according to Pan-UK Biobank, Return 2442) deemed suitable for genetic analyses (according to the criteria stated in the “Cohort selection and MRI quality control” section, above). Our missingness and Hardy-Weinberg equilibrium cutoffs are the same as those used by the flagship UK Biobank exome sequencing study^179^. We performed this quality control using version 2.0.0 of the plink GWAS software package^180^. For computational efficiency, regenie constructs the PRS using stacked ridge regression, by partitioning the genome into non-overlapping blocks of 200 markers (where 200 is specified using the “--bsize” option), running ridge regression within each block, and then running a second level of ridge regression to aggregate across blocks.

We ran step 2 on a larger set of 9,423,516 genotyped or imputed autosomal and non-pseudoautosomal X-chromosome variants with INFO score >0.8, using the same quality control filters as in step 1: minor allele frequency >1%, <10% missingness and Hardy-Weinberg equilibrium *p* > 1 × 10^–15^ (with mid-P correction). As described in the flagship UK Biobank study^9^, variants were imputed to two reference panels, the Haplotype Reference Consortium^75^ and a combined UK10K^76^ and 1000 Genomes Phase 3^77^ panel; the imputed genotypes for the two panels were then combined, using the HRC imputation for variants present in both panels.

We covaried for age (“Age when attended assessment center”, Data-Field 21003, standardized to mean 0 and variance 1), sex, age × sex, age^2^, age^2^ × sex, genotyping array (Axiom versus BiLEVE), scanner site (*n* = 22, “UK Biobank assessment center”, Data-Field 54), total intracranial volume (“Volume of EstimatedTotalIntraCranial (whole brain)”, Data-Field 26521; median-imputed for 9 people for whom it was missing), and the top 10 genotype principal components (“Genetic principal components”, Data-Field 22009) to control for population structure. Prior to running regenie, we applied a rank-based inverse normal transformation to the residuals using the Blom transformation (c = 3/8)^181^. This ensures that the GWAS phenotype is normally distributed even though the connectivity measures are highly non-normally distributed, nor are they particularly well approximated by a log-normal distribution (Supplementary Fig. 7).

Regenie assumes full dosage compensation for variants on the X chromosome (except for variants in the pseudoautosomal region, which were analyzed the same way as autosomal variants), coding alleles as 0/1/2 for females and 0/2 for males^182^; we excluded the Y chromosome.

### Identification of independent genome-wide significant variants

To identify independent genome-wide significant variants, we clumped together variants within 5 megabases and with linkage disequilibrium (LD) R^2^ > 0.01, using the --clump command from plink version 2.0.0 (https://www.cog-genomics.org/plink/2.0/postproc#clump), with options --clump-p1 5e-8 --clump-p2 0.001 --clump-r2 0.01 --clump-kb 5000. For Fig. 3, we performed this clumping on the minimum *p* value for each variant across the 206 measures (i.e. the p-values shown in the Manhattan plots). For Supplementary Data 1, we instead performed this clumping separately for each of the 206 GWAS.

### Ideogram of associated genomic loci

We plotted the ideogram of associated genomic loci in Fig. 1 with PhenoGram (http://visualization.ritchielab.org/phenograms/plot)^183^.

### *p* value inflation and linkage disequilibrium score regression intercepts and heritabilities

We observed acceptably mild *p* value inflation, with genomic inflation factors (λ_GC_) of 1.014 to 1.093 (median 1.044) across the 206 GWAS. Most of this inflation was due to polygenicity rather than confounding, with linkage disequilibrium (LD) score regression intercepts^148^ of only 0.996 to 1.016 (median 1.006).

We used the --h2 command from the ldsc software package (https://github.com/bulik/ldsc) to compute these LD score regression intercepts, as well as the heritabilities in Supplementary Fig. 2. Before running --h2, we used ldsc’s munge_sumstats.py script to reformat each GWAS’s summary statistics into the format recognized by ldsc. Rather than using predefined LD scores, we elected to compute our own LD scores for the 9,423,516 variants in our GWAS from the UK Biobank’s imputed genotypes themselves. We did so by (1) randomly subsampling 1000 of the 26,333 participants (for computational tractability), (2) converting imputed variants to hardcalls (since ldsc does not support fractional genotypes), and (3) running ldsc’s --l2 command with the UK Biobank’s hardcall genotypes provided via the --bfile flag. When running --l2, we used a 1-megabase window (--ld-wind-kb 1000) instead of the usual 1-centimorgan window (--ld-wind-cm 1), since the UK Biobank’s imputed genotype files do not report centimorgan distances.

### Heritability, polygenicity and selection analysis with SBayesS

We used the SBayesS method^147^ to estimate heritability (*h*^2^), polygenicity (*π*) and selection (*S*) parameters for each of our 206 GWAS. We ran the --sbayes S command from the Genome-wide Complex Trait Bayesian analysis (GCTB) software package (https://cnsgenomics.com/software/gctb) on each GWAS’s summary statistics, with the “Sparse matrix (including MHC regions)” from https://cnsgenomics.com/software/gctb/#LDmatrices (ukbEURu_imp_v3_HM3_n50k.chisq10.ldm.sparse) as the LD matrix and a fixed random seed of 0. SBayesS uses Markov Chain Monte Carlo (MCMC) sampling to infer *h*^2^, *π* and *S*; we used the default settings of running 10,000 iterations of the Markov chain (--chain-length 10000) and sampling the parameters from every 10th iteration (--thin 10), starting at iteration 2000 (--burn-in 2000), thus generating 800 estimates of each parameter (i.e. from iterations 2000, 2010, 2020, … 9990). We obtained a single estimate per parameter by taking the mean across the 800 estimates, and confidence intervals by taking the 2.5th and 97.5th percentiles of these 800 estimates.

### Genetic correlation analysis

We used the genetic covariance analyzer (GNOVA) method^149^ (github.com/qlu-lab/GNOVA-2.0) to compute genetic correlations between the 206 structural connectivity measures and 15 brain-related traits:

1. Major depression^184^, with summary statistics from the PGC (“mdd2019edinburgh” at https://www.med.unc.edu/pgc/download-results, under “Genome-wide summary statistics from a meta-analysis of PGC and UK Biobank”, filename PGC_UKB_depression_genome-wide.txt)
2. Bipolar disorder^185^, with summary statistics from the PGC (“bip2021” at https://www.med.unc.edu/pgc/download-results, filename pgc-bip2021-all.vcf.tsv.gz)
3. Schizophrenia^88^, with summary statistics from the PGC (“scz2022” at https://www.med.unc.edu/pgc/download-results, filenames PGC3_SCZ_wave3.primary.autosome.public.v3.vcf.tsv.gz and PGC3_SCZ_wave3.primary.chrX.public.v3.vcf.tsv.gz)
4. Autism spectrum disorder (ASD)^186^, with summary statistics from the PGC (“asd2019” at https://www.med.unc.edu/pgc/download-results, filename iPSYCHPGC_ASD_Nov2017.gz)
5. Attention-deficit hyperactivity disorder (ADHD)^187^, with summary statistics from the PGC and iPSYCH (“Summary statistics ADHD meta-analysis, Jan2022 Release” at https://ipsych.dk/en/research/downloads, filename ADHD_meta_Jan2022_iPSYCH1_iPSYCH2_deCODE_PGC.meta_2.zip)
6. Problematic alcohol use^188^, with summary statistics from the Million Veteran Program (available through the Database of Genotypes and Phenotypes (dbGaP) by application at https://www.ncbi.nlm.nih.gov/projects/gap/cgi-bin/study.cgi?study_id=phs001672.v6.p1, filename PAU_MVP1_MVP2_PGC_UKB_Dec11.txt.gz)
7. Insomnia^189^, with summary statistics from https://ctg.cncr.nl/software/summary_statistics (filename insomnia_ukb2b_EUR_sumstats_20190311_with_chrX_mac_100.txt.gz)
8. Alzheimer’s disease^190^, with summary statistics from the GWAS Catalog (accession GCST90027158, filename GCST90027158_buildGRCh38.tsv.gz)
9. Parkinson’s disease^191^, with summary statistics from https://drive.google.com/file/d/1FZ9UL99LAqyWnyNBxxlx6qOUlfAnublN (filename nallsEtAl2019_excluding23andMe_allVariants.tab.gz)
10. Amyotrophic lateral sclerosis (ALS)^192^, with summary statistics from the GWAS Catalog (accession GCST90027164, filename GCST90027164_buildGRCh37.tsv.gz)
11. Epilepsy^193^, with summary statistics from http://www.epigad.org/download/final_sumstats.zip, filename ILAE3_Caucasian_GGE_final.tbl. We used summary statistics for genetic generalized epilepsy (GGE), which had substantially greater GWAS signal than non-subtype-specific epilepsy.
12. Stroke^194^, with summary statistics from the GWAS Catalog (accession GCST90104539, filename GCST90104539_buildGRCh37.tsv.gz)
13. Educational attainment^195^, with summary statistics from https://www.thessgac.com under “Summary Statistics for Okbay et al. (2022)” (login required, filename EA4_additive_excl_23andMe.txt.gz)
14. Intelligence^196^, with summary statistics from https://ctg.cncr.nl/software/summary_statistics (filename SavageJansen_2018_intelligence_metaanalysis.txt.gz)
15. Reaction time^197^, with summary statistics from the GWAS Catalog (accession GCST006268, filename Davies2018_UKB_RT_summary_results_29052018.txt). The sign of the GWAS beta coefficients are flipped in this GWAS, so that a positive beta indicates that a variant is associated with lower (i.e. better) reaction time, and a negative beta indicates that a variant is associated with higher (i.e. worse) reaction time.

To account for case-control imbalance in analyses of case-control GWAS, the effective sample size *N*_*eff*_  = 4 / (1 / *N*_*cas*_ + 1 / *N*_*con*_) can be used as a measure of sample size, where *N*_*cas*_ and *N*_*con*_ are either total number of cases and controls in the GWAS, or (more accurately) the number of cases and controls with non-missing genotypes for each variant. For meta-analyses of multiple GWAS cohorts, it has been argued that *N*_*eff*_ should be computed per cohort and then summed across cohorts^198^, since each cohort has a different case-control ratio. Unfortunately, most publicly available case-control summary statistics either do not provide *N*_*eff*_ or compute it using the total *N*_*cas*_ and *N*_*con*_ instead of the per-cohort *N*_*cas*_ and *N*_*con*_, which can lead to substantial bias in estimates of heritability (though not necessarily genetic correlation)^198^. In these cases, we inferred *N*_*eff*_ for each variant using the formula N_eff_ = ((4 / (2 × *MAF* × (1 - *MAF*) × *INFO*)) - *BETA*^*2*^) / *SE*^2^, where *MAF* is the variant’s minor allele frequency in the GWAS sample, *INFO* its imputation information score, *BETA* its effect size, and *SE* the standard error of this effect size^199^. If *INFO* was not provided, we excluded the *INFO* term. If *SE* was not provided, we back-calculated it using the variant’s effect size and *p* value. If *MAF* was not provided (or was only provided for a different sample, such as 1000 Genomes), we fell back to using each variant’s total *N*_*cas*_ and *N*_*con*_ across all cohorts (or, if per-variant *N*_*cas*_ and *N*_*con*_ were not available, the total *N*_*cas*_ and *N*_*con*_ across all cohorts) and calculated *N*_*eff*_ as 4 / (1 / *N*_*cas*_ + 1 / *N*_*con*_). For our own structural connectivity GWASs and other quantitative-trait GWASs (e.g. reaction time), we did not compute an effective sample size and simply used the total sample size.

We harmonized the alleles of the 15 summary statistics, as well as our own GWAS summary statistics, to the European subset of 1000 Genomes Phase 3^77^ (storage.googleapis.com/broad-alkesgroup-public/LDSCORE/1000G_Phase3_plinkfiles.tgz) using ldsc’s munge_sumstats.py script. We then removed variants with missing data so that the munged summary statistics would be compatible with GNOVA. Finally, we ran GNOVA to compute genetic correlations, specifying the European subset of 1000 Genomes Phase 3 as a reference panel for computing LD scores.

We also used GNOVA to perform genetic correlations between our three hemisphere-level measures and 432 GWAS of diffusion MRI measures based on tract-based spatial statistics (TBSS) and neurite orientation dispersion and density imaging (NODDI)^18^. We obtained summary statistics for these GWAS from the table at open.win.ox.ac.uk/ukbiobank/big40/BIG40-IDPs_v4/IDPs.html, subsetting to the 432 diffusion MRI measures in the UK Biobank’s “dMRI skeleton” phenotype category (Category 134; biobank.ctsu.ox.ac.uk/crystal/label.cgi?id=134). We followed the same procedure as for the 15 summary statistics above, using the N(all) column in each summary statistics file as the sample size.

### Cell-type enrichment analysis

We used partitioned LD score regression^151^ from the ldsc software package to perform cell-type enrichment analyses across human cortical cell types. We used two chromatin accessibility datasets, one adult and one developmental.

First, we included regions for 6 cell types from postmortem adult human motor cortex, derived from single-nucleus chromatin accessibility and messenger RNA expression sequencing (SNARE-seq2). We obtained these regions from Table S14c of ^152^: “SNARE-Seq2 Differentially Accessible Regions (DARs, q value < 0.001 and log(fold change) > 1) identified by subclass for human M1”. Since these regions were only available for neuronal subclasses, but a subclass-specific analysis would likely be underpowered, we merged regions across subclasses. We obtained excitatory neuron-specific regions by merging regions specific to layer 2–3 intratelencephalic (L2-3 IT), layer 5 extratelencephalic (L5 ET), layer 5 intratelencephalic (L5 IT), layer 5-6 non-projecting pyramidal (L5-6 NP), layer 6 corticothalamic (L6 CT), layer 6 intratelencephalic (L6 IT), *CAR3*-expressing layer 6 intratelencephalic (L6 IT Car3), and layer 6b (L6b) excitatory neurons. We obtained inhibitory neuron-specific regions by merging regions specific to LAMP5, PVALB, SNCG, SST, SST CHODL, and VIP inhibitory neurons. We note that this choice does not capture excitatory neuron-specific regions that are present in multiple excitatory neuron subclasses, or inhibitory neuron-specific regions that are present in multiple inhibitory neuron subclasses. Besides excitatory and inhibitory, we included the following non-neuronal cell types in our analysis: astrocytes; microglia and perivascular macrophages (Micro-PVM), which we refer to as ‘microglia’ for simplicity; oligodendrocytes (Oligo); and oligodendrocyte progenitor cells (OPC). We did not include regions specific to vascular leptomeningeal cells (VLMCs) and endothelial cells due to their rarity. Thus, we included a total of 6 cell types in the analysis.

Second, we included regions for 12 cell types from the developing human cortex, derived from single-cell assay for transposase-accessible chromatin with high-throughput sequencing (ATAC-seq). We obtained these regions from Table S2 of ^154^.

We created a binary annotation file for each of these 18 cell types, with a 1 or 0 for each variant depending on whether or not it was situated in a region with increased chromatin accessibility in that cell type. For instance, for the SNARE-Seq2 dataset, 29,398 of the 9,423,516 variants in our GWAS (0.35%) were situated in astrocyte-specific open chromatin regions, 112,321 (1.33%) in excitatory-specific regions, 87,192 (1.03%) in inhibitory-specific regions, 35,754 (0.42%) in microglia-specific regions, 30,394 (0.36%) in oligodendrocyte-specific regions, and 36,246 (0.43%) in OPC-specific regions. We also created an “intercept” annotation file in which all of the 9,423,516 variants were assigned a 1.

We computed LD scores for each of these binary annotation files by applying ldsc’s --l2 command with a 1-megabase window (--ld-wind-kb 1000), providing the UK Biobank’s hardcall genotypes with the --bfile flag, and providing the annotation file with the --annot and --thin-annot flags. Aside from providing an annotation file, this is the same way we computed LD scores for all variants (see the “*p* value inflation and linkage disequilibrium score regression intercepts” subsection, above). Note that the LD scores for the intercept annotation are the same as the LD scores for all variants.

Finally, for each of the 206 structural connectivity GWAS and each of the 18 cell types, we ran stratified LD score regression using ldsc’s --h2 command. We provided the GWAS’s summary statistics under the --h2 flag, the LD scores for both the intercept and the cell type under the --ref-ld flag, the LD scores for the intercept under the --w-ld flag, and the allele frequencies for each variant across our 26,333 participants (computed with plink 1.9’s --freq command) under the --frqfile flag. We also specified the --overlap-annot flag to indicate that the two annotations (for the intercept and the cell type) overlap with each other, and the --print-coefficients flag to print the regression coefficients for each annotation.

This analysis yields an enrichment (and a standard error and *p* value for this enrichment) for each GWAS and cell type. This enrichment represents how many times greater heritability is explained by the average variant overlapping a region with increased chromatin accessibility in that cell type, relative to the average variant not overlapping such a region. The use of LD score regression makes this analysis robust to the confounding effects of LD: without it, an annotation could erroneously be considered enriched for heritability even if the entirety of the GWAS signal underlying this enrichment were coming from variants in LD not overlapping the annotation.

### Reporting summary

Further information on research design is available in the Nature Portfolio Reporting Summary linked to this article.

### Supplementary information

Supplementary Information

Description of Additional Supplementary Files

Supplementary Data 1

Supplementary Data 2

Supplementary Data 3

Supplementary Data 4

Reporting Summary

## Data Availability

Researchers can apply for access to the UK Biobank at ukbiobank.ac.uk/enable-your-research/apply-for-access. Genome-wide summary statistics have been deposited to the European Bioinformatics Institute GWAS Catalog (https://www.ebi.ac.uk/gwas) under accession numbers GCST90302648 through GCST90302853. The 206 structural connectivity measures for each participant will be made available through the UK Biobank Returns Catalog (biobank.ndph.ox.ac.uk/ukb/docs.cgi?id=1) to all researchers with UK Biobank access.

## Code availability

The tractography pipeline used in this study, tractify, is available at https://github.com/TIGRLab/tractify.
