# Supplementary material for: Genetic architecture of the structural connectome
Source: Nat Commun. 2024 Mar 4;15:1962. doi: 10.1038/s41467-024-46023-2 (PMC10912129; doi:10.1038/s41467-024-46023-2)
Supplement: Supplementary file 1 — Supplementary Information [file 41467_2024_46023_MOESM1_ESM.pdf]

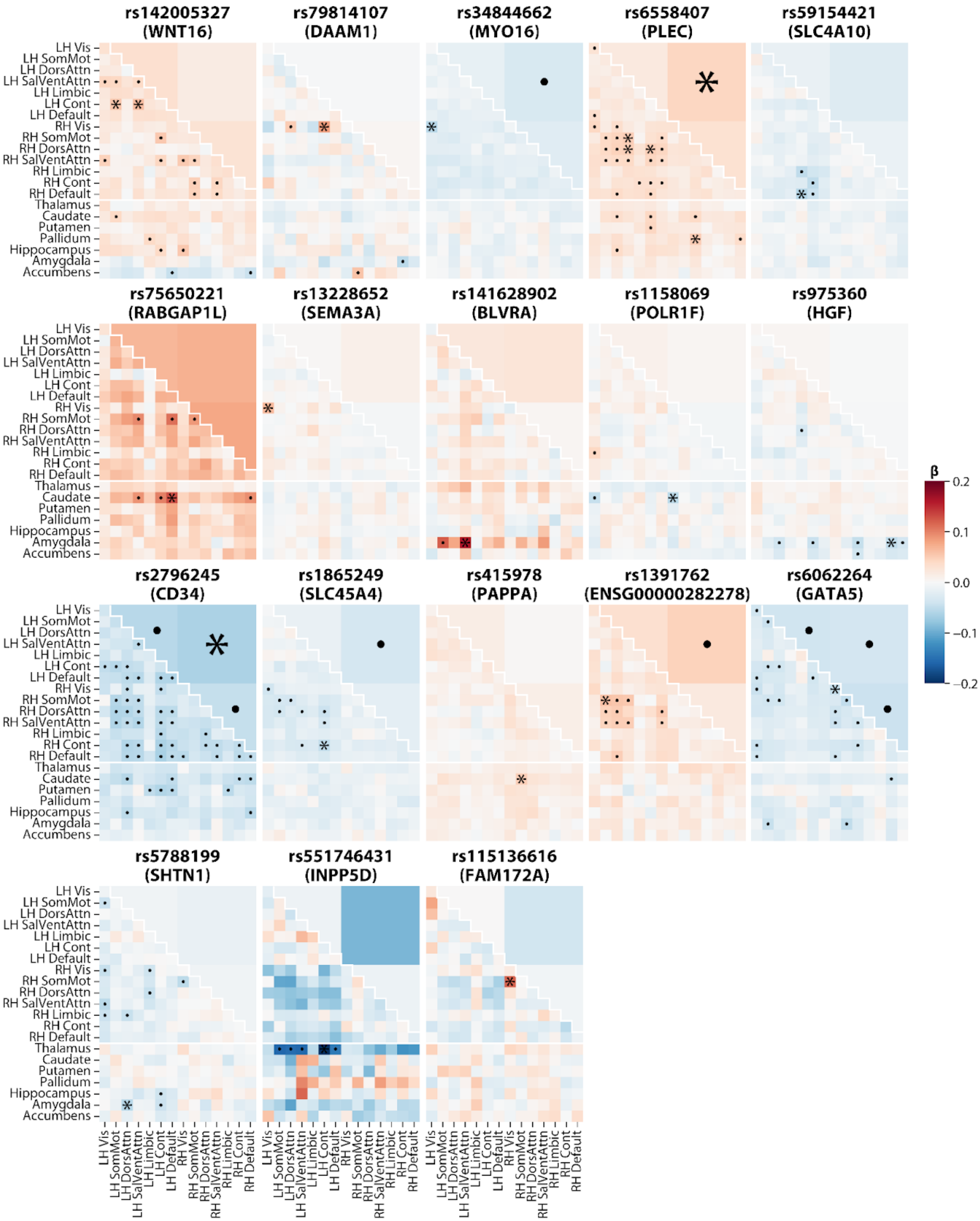

Supplementary Figure 1: Spatial patterns of association with structural connectivity. Continuation of Figure 4. rs59154421 (*SLC4A10*) is referred to by its UK Biobank variant identifier 2:162813034_CTG_C in the Supplementary Data and summary statistics, while rs551746431 (*INPP5D*) is referred to by its UK Biobank ID 2:234112457_CCAGCTACTCCCAAGTAGTCT_C.

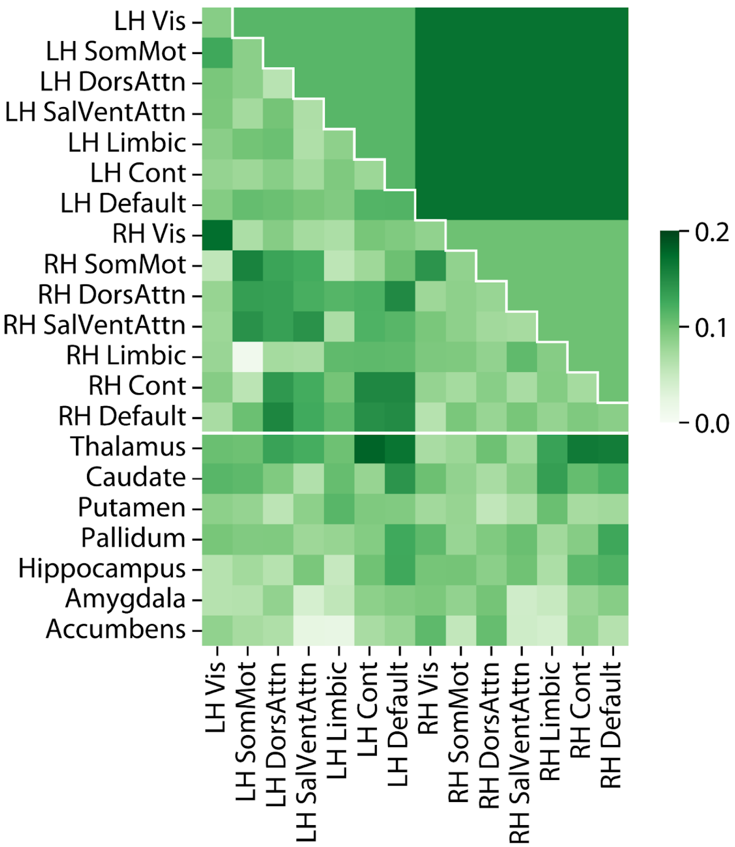

Supplementary Figure 2: Heritability of each of the 206 measures according to LD score regression.

Supplementary Figure 3 (next page): Genetic correlation between structural connectivity measures and 15 brain-related traits. Asterisks (*) indicate significantly positive (red) or negative (blue) genetic correlations, after Bonferroni correction for the 206 × 15 genetic correlations tested. ADHD = attention-deficit hyperactivity disorder, ALS = amyotrophic lateral sclerosis, LH = left-hemisphere, RH = right-hemisphere, Vis = visual, SomMot = somatomotor, DorsAttn = dorsal attention, SalVentAttn = salience/ventral attention, Limbic = limbic, Cont = control, Default = default mode.

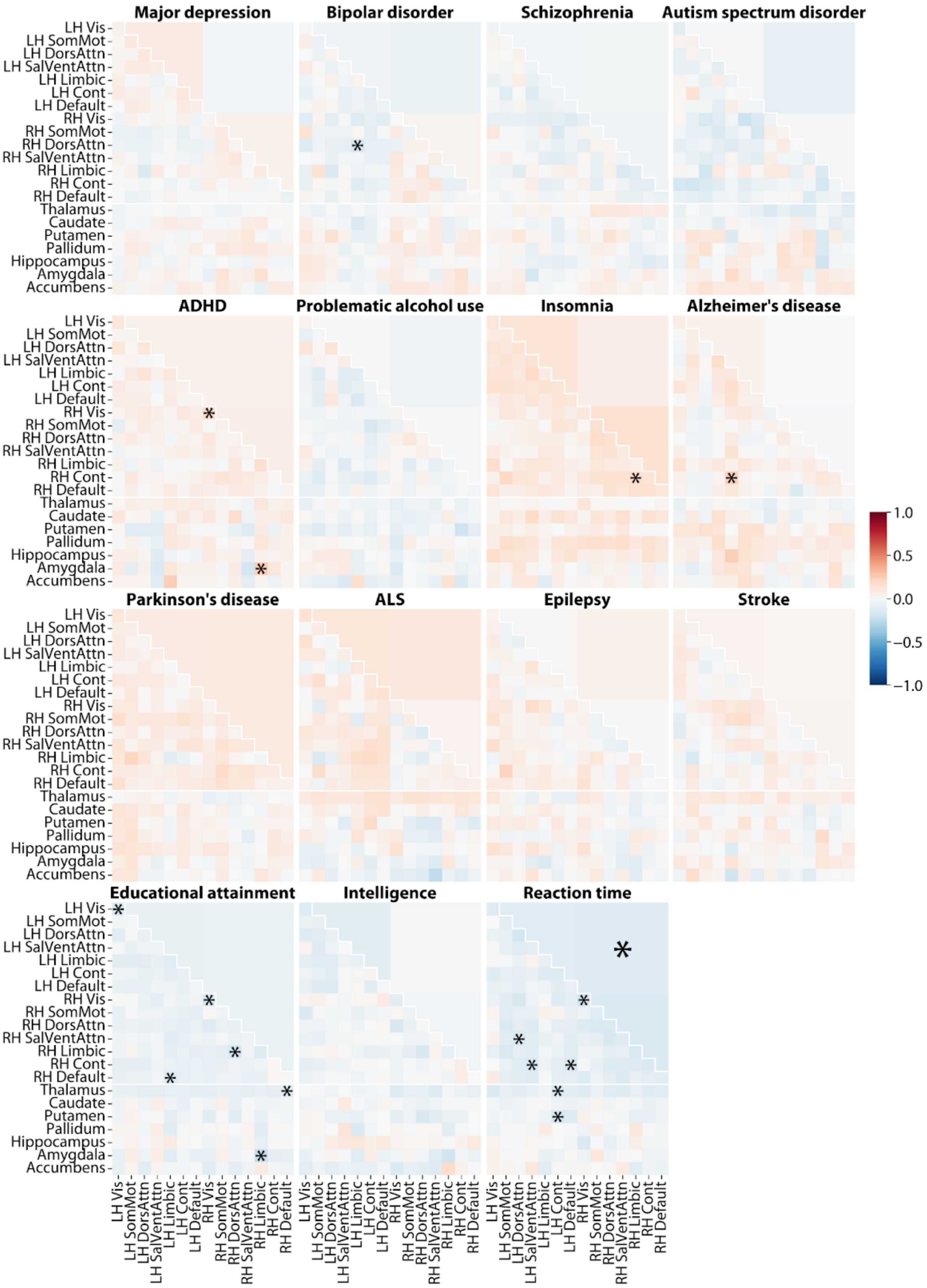
Major depression
Bipolar disorder
Schizophrenia
Autism spectrum disorder
ADHD
Problematic alcohol use
Insomnia
Alzheimer's disease
Parkinson's disease
ALS
Epilepsy
Stroke
Educational attainment
Intelligence
Reaction time
LH Vis
LH SomMot
LH DorsAttn
LH SalVentAttn
LH Limbic
LH Cont
LH Default
RH Vis
RH SomMot
RH DorsAttn
RH SalVentAttn
RH Limbic
RH Cont
RH Default
Thalamus
Caudate
Putamen
Pallidum
Hippocampus
Amygdala
Accumbens
1.0
0.5
0.0
−0.5
−1.0

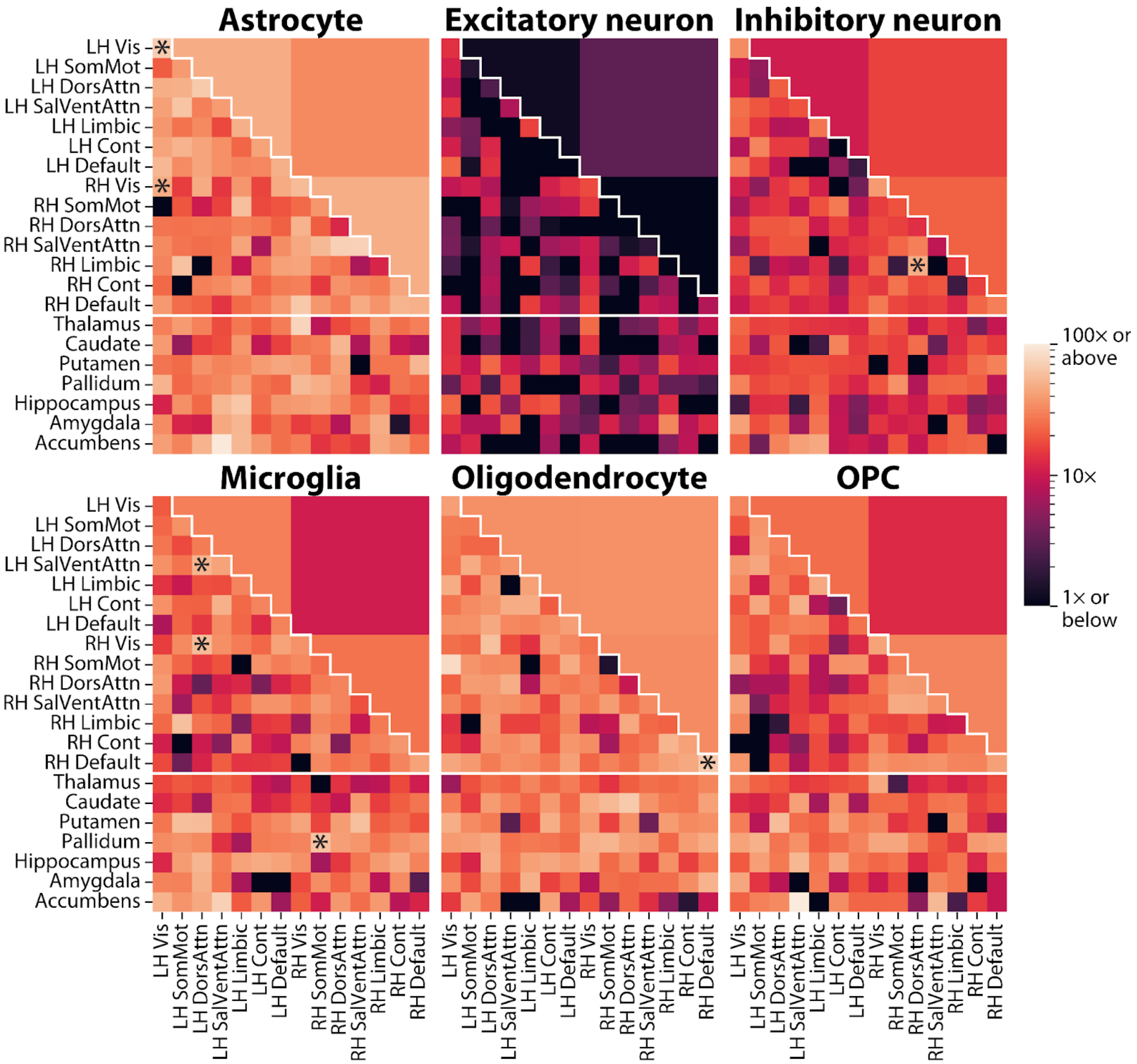

Supplementary Figure 4: Heritability enrichment for 6 adult brain cell types. Asterisks (*) indicate significant heritability enrichments of structural connectivity measures for adult brain cell types after Bonferroni correction for the 206 × 6 cell-type enrichments tested. OPC = oligodendrocyte precursor cell, LH = left-hemisphere, RH = right-hemisphere, Vis = visual, SomMot = somatomotor, DorsAttn = dorsal attention, SalVentAttn = salience/ventral attention, Limbic = limbic, Cont = control, Default = default mode.

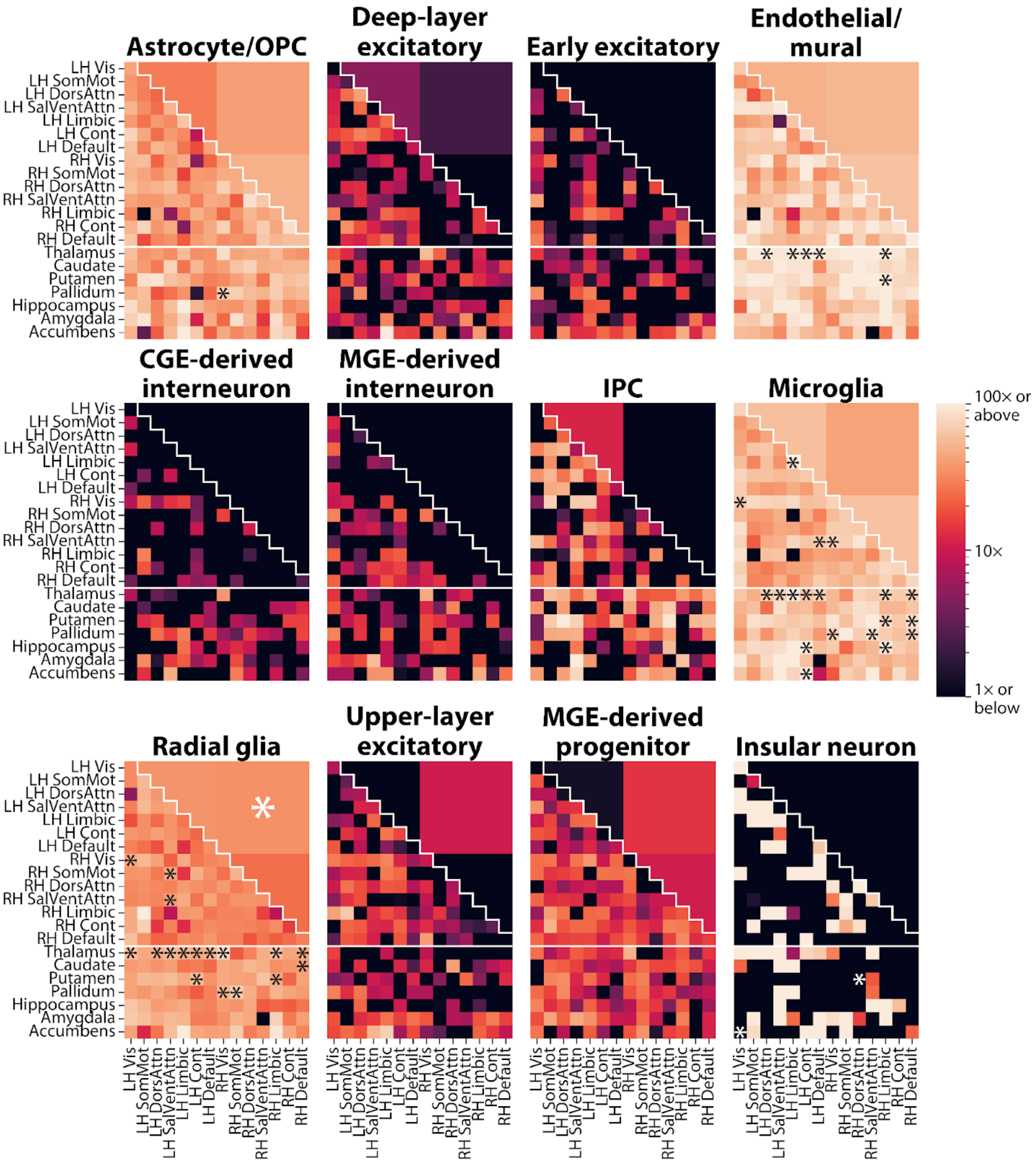

Supplementary Figure 5: Heritability enrichment for 12 developmental brain cell types. Asterisks (*) indicate significant heritability enrichments of structural connectivity measures for developmental brain cell types after Bonferroni correction for the 206 × 12 cell-type enrichments tested. OPC = oligodendrocyte precursor cell, excitatory = excitatory neuron, CGE = caudal ganglionic eminence, MGE = medial ganglionic eminence, IPC = intermediate progenitor cell, LH = left-hemisphere, RH = right-hemisphere, Vis = visual, SomMot = somatomotor, DorsAttn = dorsal attention, SalVentAttn = salience/ventral attention, Limbic = limbic, Cont = control, Default = default mode.

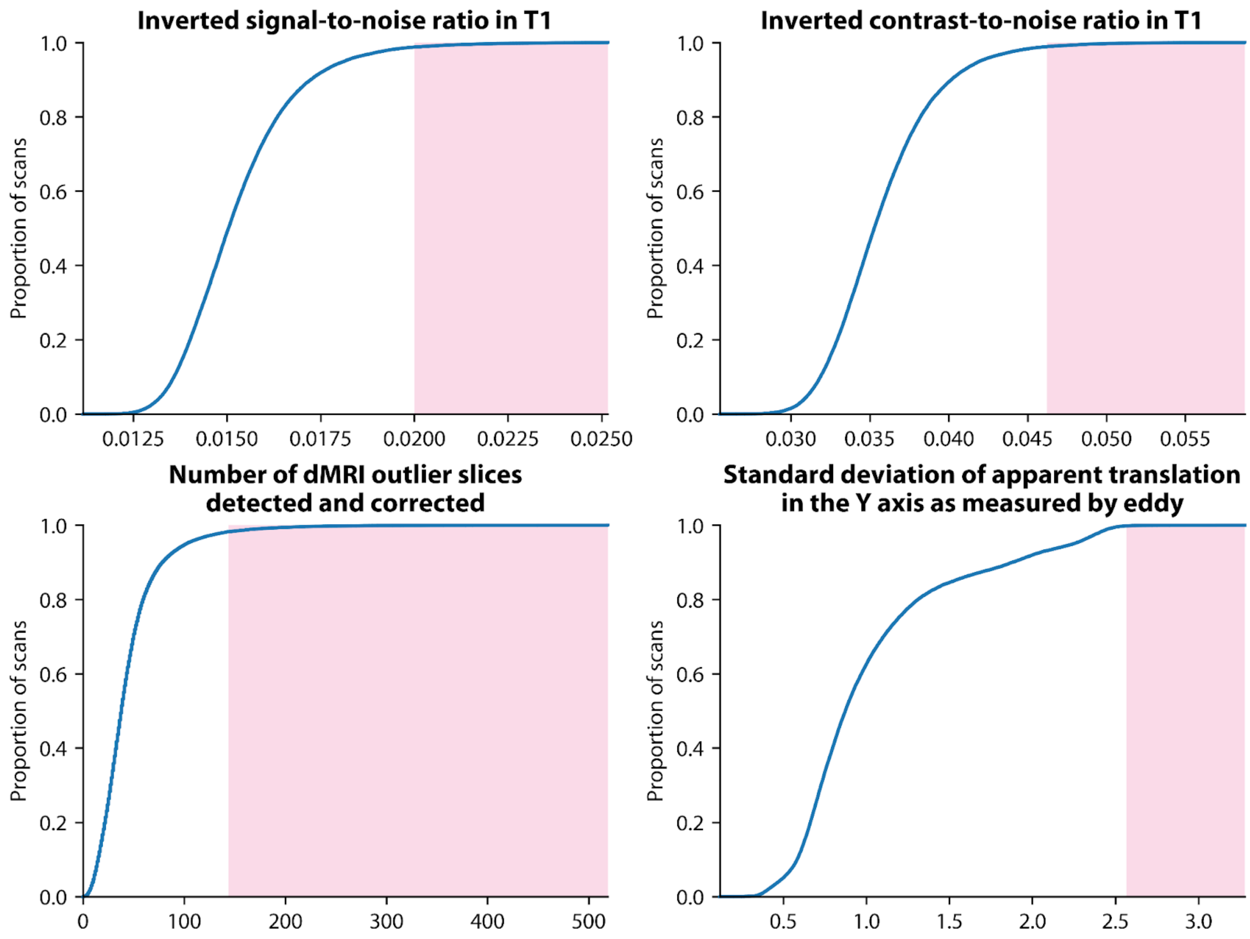

Supplementary Figure 6: Cumulative distribution plots of the four metrics used for quality control, across all 31,309 pre-quality control baseline scans. Scans where any of the four metrics were more than 3 standard deviations above the mean (red regions) were excluded.

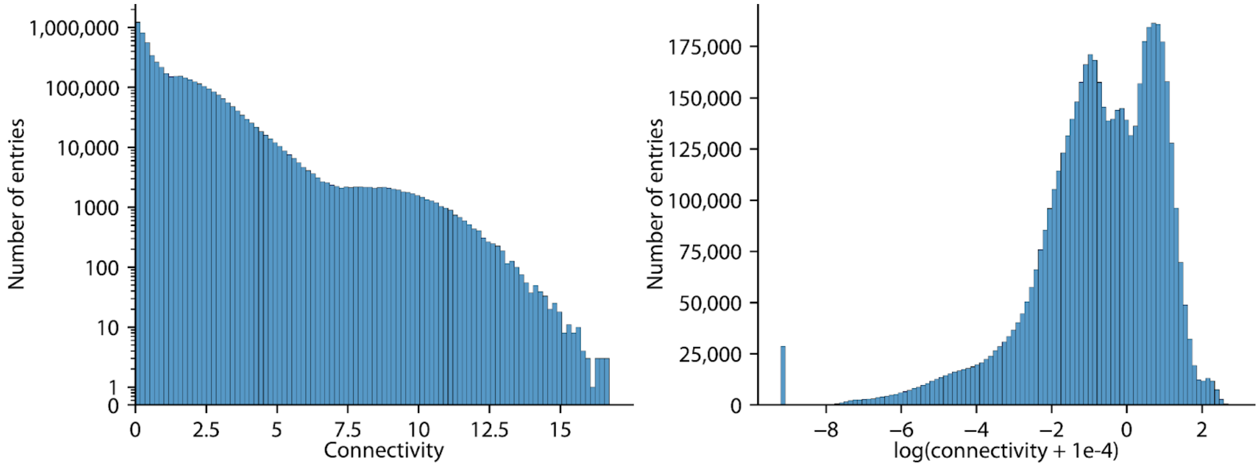

Supplementary Figure 7: Histogram of entries in the 26,333-participant-by-206-measure structural connectivity matrix, both on the original scale (left) and log-transformed (right). The averages of the columns of this matrix (i.e. the averages of each of the measures across participants) are shown in Figure 2B. Note the logarithmic scale of the y-axis in the left panel.
