# Supplementary material for: Genetic architecture of the structural connectome
Source: Nat Commun. 2024 Mar 4;15:1962. doi: 10.1038/s41467-024-46023-2 (PMC10912129; doi:10.1038/s41467-024-46023-2)
Supplement: Supplementary file 2 — Description of Additional Supplementary Files [file 41467_2024_46023_MOESM2_ESM.pdf]

**File name: Supplementary Data 1**
**Description: Lead variants associated with each connectivity measure at nominal genome-wide significance ($p < 5 \times 10^{-8}$).** The “Clump start” and “Clump end” columns indicate the extent of all variants in the lead variant’s linkage disequilibrium clump (see “Identification of independent genome-wide significant variants”, Methods). The “Nearest gene(s)” column indicates the nearest gene to the lead variant, or a comma-separated list of nearest genes if there are multiple. The “Bonferroni-significant” column indicates associations passing Bonferroni-corrected genome-wide significance ($p < 5 \times 10^{-8} / 206$). The “Minimum p-value at its locus?” column indicates whether the lead variant has the minimum p-value at its locus across all connectivity measures; these variants are highlighted with black diamonds in Figure 3. Base pair positions are in hg19 coordinates.

**File name: Supplementary Data 2**
**Description: Replication analysis in 665 participants of non-European genetic ancestry.** 75 of the 126 lead variants from the primary analysis had at least 1% frequency and passed quality control, and are shown in the table.

**File name: Supplementary Data 3**
**Description: Phenotypic and genetic correlations between the three hemisphere-level measures and 432 prior GWAS of diffusion MRI measures.** Phenotypic correlations and p-values were calculated via Pearson correlation, while genetic correlations and p-values were calculated using GNOVA. P-values are two-sided and uncorrected for multiple comparisons.

**File name: Supplementary Data 4**
**Description: The 214-parcel atlas used to create each participant’s connectome matrix.** We concatenated the 200-parcel Schaefer cortical atlas, mapped to the “Yeo 7” networks, with 14 subcortical parcels from the Harvard-Oxford atlas: left thalamus, left caudate, left putamen, left pallidum, left hippocampus, left amygdala, left accumbens, right thalamus, right caudate, right putamen, right pallidum, right hippocampus, right amygdala, and right accumbens.
