# Supplementary material for: Genetic architecture of the structural connectome
Source: Nat Commun. 2024 Mar 4;15:1962. doi: 10.1038/s41467-024-46023-2 (PMC10912129; doi:10.1038/s41467-024-46023-2)
Supplement: Supplementary file 7 — Reporting Summary [file 41467_2024_46023_MOESM7_ESM.pdf]

nature portfolio

Corresponding author(s): Michael Wainberg, Colin Hawco, Shreejoy Tripathy

Last updated by author(s): Nov 22, 2023

Reporting Summary

Nature Portfolio wishes to improve the reproducibility of the work that we publish. This form provides structure for consistency and transparency in reporting. For further information on Nature Portfolio policies, see our Editorial Policies and the Editorial Policy Checklist.

Statistics

For all statistical analyses, confirm that the following items are present in the figure legend, table legend, main text, or Methods section.

n/a	Confirmed	
☐	☒	The exact sample size (n) for each experimental group/condition, given as a discrete number and unit of measurement
☐	☒	A statement on whether measurements were taken from distinct samples or whether the same sample was measured repeatedly
☐	☒	The statistical test(s) used AND whether they are one- or two-sided<br>*Only common tests should be described solely by name; describe more complex techniques in the Methods section.*
☐	☒	A description of all covariates tested
☐	☒	A description of any assumptions or corrections, such as tests of normality and adjustment for multiple comparisons
☐	☒	A full description of the statistical parameters including central tendency (e.g. means) or other basic estimates (e.g. regression coefficient) AND variation (e.g. standard deviation) or associated estimates of uncertainty (e.g. confidence intervals)
☐	☒	For null hypothesis testing, the test statistic (e.g. F, t, r) with confidence intervals, effect sizes, degrees of freedom and P value noted<br>*Give P values as exact values whenever suitable.*
☒	☐	For Bayesian analysis, information on the choice of priors and Markov chain Monte Carlo settings
☒	☐	For hierarchical and complex designs, identification of the appropriate level for tests and full reporting of outcomes
☐	☒	Estimates of effect sizes (e.g. Cohen's d, Pearson's r), indicating how they were calculated

Our web collection on statistics for biologists contains articles on many of the points above.

Software and code

Policy information about availability of computer code

Data collection	This study used the UK Biobank. No new data were collected.
Data analysis	Our tractography pipeline, tractify (https://github.com/TIGRLab/tractify), performs probabilistic tractography using version 3.0.3 of the MRtrix3 diffusion MRI software package. Certain steps also use version 6.0.5 of the Functional Magnetic Resonance Imaging of the Brain Software Library (FSL) and version 7.1.1 of the FreeSurfer software package. We performed genome-wide association studies using version 3.2.9 of the regenie genetic analysis toolkit (https://rgcgithub.github.io/regenie). We performed GWAS quality control with version 2.0.0 of the plink software package (https://www.cog-genomics.org/plink/2.0).

For manuscripts utilizing custom algorithms or software that are central to the research but not yet described in published literature, software must be made available to editors and reviewers. We strongly encourage code deposition in a community repository (e.g. GitHub). See the Nature Portfolio guidelines for submitting code & software for further information.

Data

Policy information about availability of data

All manuscripts must include a data availability statement. This statement should provide the following information, where applicable:

- Accession codes, unique identifiers, or web links for publicly available datasets
- A description of any restrictions on data availability
- For clinical datasets or third party data, please ensure that the statement adheres to our policy

Researchers can apply for access to the UK Biobank at ukbiobank.ac.uk/enable-your-research/apply-for-access. Connectivity matrices for the structural connectomes analyzed in this study will be made available through the UK Biobank Returns Catalogue (biobank.ndph.ox.ac.uk/ukb/docs.cgi?id=1) to all researchers with UK Biobank access.

Research involving human participants, their data, or biological material

Policy information about studies with human participants or human data. See also policy information about sex, gender (identity/presentation), and sexual orientation and race, ethnicity and racism.

Reporting on sex and gender	Sex was included as a covariate.
Reporting on race, ethnicity, or other socially relevant groupings	Genetic ancestry was used for cohort stratification.
Population characteristics	Participants were 53% female and aged 40-70 (median 55) at the time of their first scan.
Recruitment	Participants were recruited by the UK Biobank as described previously
Ethics oversight	UK Biobank, Centre for Addiction and Mental Health

Note that full information on the approval of the study protocol must also be provided in the manuscript.

Field-specific reporting

Please select the one below that is the best fit for your research. If you are not sure, read the appropriate sections before making your selection.

☒ Life sciences ☐ Behavioural & social sciences ☐ Ecological, evolutionary & environmental sciences

For a reference copy of the document with all sections, see nature.com/documents/nr-reporting-summary-flat.pdf

Life sciences study design

All studies must disclose on these points even when the disclosure is negative.

Sample size	The entire UK Biobank cohort was used, after subsetting by genetic ancestry and excluding data for reasons of quality control.
Data exclusions	Participants were excluded if they lacked diffusion-weighted MRI scans corrected for eddy currents, head motion, outlier slices, and gradient distortion; lacked eddy-corrected bvec files; lacked FreeSurfer parcellation images; were more than 3 standard deviations above the mean for any of T1 inverse signal-to-noise ratio ("Inverted signal-to-noise ratio in T1", Data-Field 25734), T1 inverse contrast-to-noise-ratio ("Inverted contrast-to-noise ratio in T1", Data-Field 25735), number of diffusion MRI outlier slices ("Number of dMRI outlier slices detected and corrected", Data-Field 25746), and left-to-right head motion as measured by eddy in the diffusion MRI ("Standard deviation of apparent translation in the Y axis as measured by eddy", Data-Field 25922); or did not have genotyping data deemed suitable for genetic analyses: genotypes available, no mismatch between genetic sex ("Genetic sex", Data-Field 22001) and self-reported sex ("Sex", Data-Field 31), no sex chromosome aneuploidy ("Sex chromosome aneuploidy", Data-Field 22019), and not flagged as "Outliers for heterozygosity or missing rate" (Data-Field 22027)
Replication	Replication was performed in UK Biobank participants of non-European genetic ancestry. We identified 126 loci (Figure 1, Figure 3, Supplementary Data 1) at the less stringent threshold of nominal genome-wide significance ($p < 5 \times 10\text{-}8$). In a replication analysis of these 126 loci in 665 participants of non-European genetic ancestry, 75 of the 126 lead variants had at least 1% frequency and passed quality control, and these variants were 2.7 times more likely to have the same direction of effect than expected by chance (Fisher $p = 0.038$; Supplementary Data 2).
Randomization	Participants were not randomized. We covaried for age, sex, age × sex, age2, age2 × sex, genotyping array, scanner site (n = 22 sites), total intracranial volume, and the top 10 genotype principal components.
Blinding	Not applicable to genetic studies of brain structure because there is nothing to be blinded to.

Reporting for specific materials, systems and methods

We require information from authors about some types of materials, experimental systems and methods used in many studies. Here, indicate whether each material, system or method listed is relevant to your study. If you are not sure if a list item applies to your research, read the appropriate section before selecting a response.

Materials & experimental systems

n/a	Involved in the study
☒	☐ Antibodies
☒	☐ Eukaryotic cell lines
☒	☐ Palaeontology and archaeology
☒	☐ Animals and other organisms
☒	☐ Clinical data
☒	☐ Dual use research of concern
☒	☐ Plants

Methods

n/a	Involved in the study
☒	☐ ChIP-seq
☒	☐ Flow cytometry
☒	☐ MRI-based neuroimaging

Plants

Seed stocks	*Report on the source of all seed stocks or other plant material used. If applicable, state the seed stock centre and catalogue number. If plant specimens were collected from the field, describe the collection location, date and sampling procedures.*
Novel plant genotypes	*Describe the methods by which all novel plant genotypes were produced. This includes those generated by transgenic approaches, gene editing, chemical/radiation-based mutagenesis and hybridization. For transgenic lines, describe the transformation method, the number of independent lines analyzed and the generation upon which experiments were performed. For gene-edited lines, describe the editor used, the endogenous sequence targeted for editing, the targeting guide RNA sequence (if applicable) and how the editor was applied.*
Authentication	*Describe any authentication procedures for each seed stock used or novel genotype generated. Describe any experiments used to assess the effect of a mutation and, where applicable, how potential secondary effects (e.g. second site T-DNA insertions, mosiacism, off-target gene editing) were examined.*
